# Time Out: A Scoping Review of Non‐Duration Based Social Media Use Measures and Adolescent Mental Health

**DOI:** 10.1002/jad.70088

**Published:** 2025-12-19

**Authors:** Amanda M. Sursely, Bengi Baran, Gerta Bardhoshi, Jonathan M. Platt

**Affiliations:** ^1^ Department of Epidemiology College of Public Health, University of Iowa Iowa City Iowa USA; ^2^ Department of Psychological and Brain Sciences University of Iowa Iowa City Iowa USA; ^3^ Department of Rehabilitation and Counselor Education University of Iowa Iowa City Iowa USA

**Keywords:** adolescent, depression, mental health, social media, social networking sites, youth

## Abstract

**Introduction:**

Research to understand the role of social media use (SMU) in explaining deteriorating adolescent mental health has been limited by broad, nonspecific measures of social media use, specifically ‘time spent on social media’. These measures provide insufficient detail to capture specific risk and protective factors to users.

**Methods:**

We conducted a scoping review of observational and experimental studies of the relationship between non‐duration‐based SMU measures and mental health outcomes in adolescents ≤ 18 years old. Studies that measured SMU solely based on time spent on a platform were excluded.

**Results:**

The initial search returned 868 articles. After inclusion and exclusion, we identified 217 studies, but among them 133 (61%) used duration‐based SMU measures and were excluded. Of the 84 remaining studies, most focused on depression (48%), or anxiety (23%), though nine total mental health domains were included. Studies used 85 distinct measures of SMU, and fewer than half (*n* = 37; 45%) provided evidence of validity. SMU measures were grouped into five domains, including SMU habits, addiction‐like measures, structural aspects, interactions on SM, and feelings about SM. Social comparison and addiction‐related measures were consistently linked with poor mental health. SMU for socialization was consistently associated with decreased loneliness. Evidence of protective associations were otherwise limited.

**Conclusions:**

These findings contribute to a more complete understanding of specific types of SMU that contribute to adolescent mental health. This specificity may help to identify modifiable targets for use in prevention programs and policy development for social media regulation.

## 1 Introduction

Young people have experienced rising rates of mental health problems over the past several decades (Schrijvers et al. 2024). Increases in psychological distress, depression (Keyes et al. 2019), anxiety (Collishaw 2015), attention‐deficit hyperactivity disorder (ADHD) (Xu et al. 2018), and suicidal behaviors (Keyes and Platt 2023) have recently led the American Academy of Pediatrics, the American Academy of Child and Adolescent Psychiatrists, and the Children's Hospital Association to declare a state of national emergency (Andreassen et al. 2012). Importantly, rising rates of emergency room visits for self‐injury and death from suicide (Suicide Mortality in the United States 2023; Mercado et al. 2017; Anderson et al. 2023) suggest that trends reflect a generational increase in burden, and cannot be solely explained by changes in reporting methods and behaviors (Corredor‐Waldron and Currie 2024).

Though adolescent mental health problems have a complex etiology and reported increases cannot be attributed solely to one factor, social media use (SMU), which has rapidly become a central and nearly ubiquitous part of the social environment of youth, has received a lot of attention. Nearly all (97%) US adolescents use at least one social media platform, where they spend an average of 3 h per day. A third of adolescents say that they engage with social media “almost constantly” (Vogels et al. 2022). Peer social relationships grow in size, importance, complexity, and time‐intensiveness (Gerwin et al. 2018; Shifflet‐Chila et al. 2016; Nelson et al. 2016) during adolescence, however, the social media may distort or highlight negative aspects of social experiences, and negatively impact wellbeing by fueling anxiety, depression, loneliness, FOMO (fear of missing out), and other psychosocial stressors that are especially salient in youth (Valkenburg et al. 2022; Hancock et al. 2022).

There is an extensive body of research to understand the role of SMU as a potential cause of deteriorating adolescent mental health. To date, individual studies have been summarized through numerous narrative reviews (Bozzola et al. 2022; McCrory et al. 2020; Vidal et al. 2020; Verduyn et al. 2017), systematic reviews (Damodar et al. 2021; Keles et al. 2019; McCrae et al. 2017; Memon et al. 2018; Radtke et al. 2022; Sedgwick et al. 2019), reviews of reviews (Valkenburg et al. 2022), meta‐analyses (Hancock et al. 2022; Ivie et al. 2020), and meta‐analyses of reviews (Appel et al. 2020; Meier and Reinecke 2020). Despite this scholarship, our understanding of the significance and magnitude of SMU risk is limited by a lack of attention to how SMU is conceptualized and measured. Typical measures focus on broad contextual indicators, especially duration of time spent on social media. While important, these measures provide insufficient detail to capture unique risk and protective factors that a user may be exposed to. By analogy, a study of school‐based risk for student mental health based on ‘time spent’ at school would provide obviously crude results, likely resulting in a body of research as inconsistent as the current literature base for social media (Keyes and Platt 2024). Alongside the collection of better and more detailed measures of social media use (Corredor‐Waldron et al. 2024), there is a parallel need to review the existing literature with a greater focus on delineating relationships between specific aspects of social media use and youth mental health. Both approaches will help to identify actionable and effective policy targets and reduce harmful SMU in young people and begin to reverse the current mental health crisis they are facing. To these ends, the aims of this study were to conduct a scoping review to 1) document research on non‐duration‐based measures of social media use and mental health outcomes in youth and 2) summarize variation in the associations between SMU measures and mental health.

## 2 Methods

### 2.1 Search Strategy and Selection Criteria

This review was conducted and reported according to the guidelines established by PRISMA for Scoping Reviews (Appendix 1) (Tricco et al. 2018). The literature on social media and mental health is broad and large. Thus, to increase the efficiency of the search, a systematic literature search was first performed to identify existing systematic reviews exploring the association between social media use and adolescent mental health. Electronic databases including PubMed, Embase, and PsycINFO were searched for all relevant systematic reviews published in peer‐reviewed journals from January 2006 (the first year that Facebook was widely available) to September 2024 using a combination of search terms related social media, mental health, adolescents, and systematic review (Appendix 2). We then examined the reference lists of the resulting 45 relevant published reviews and meta‐analyses. The primary articles cited in each applicable systematic review were then extracted and reviewed for inclusion. The reference lists of these studies were also examined. To validate the completeness of this search strategy, an additional search was conducted in Google Scholar, with 100 titles reviewed to identify potential missed primary publications.

**Figure 1:**
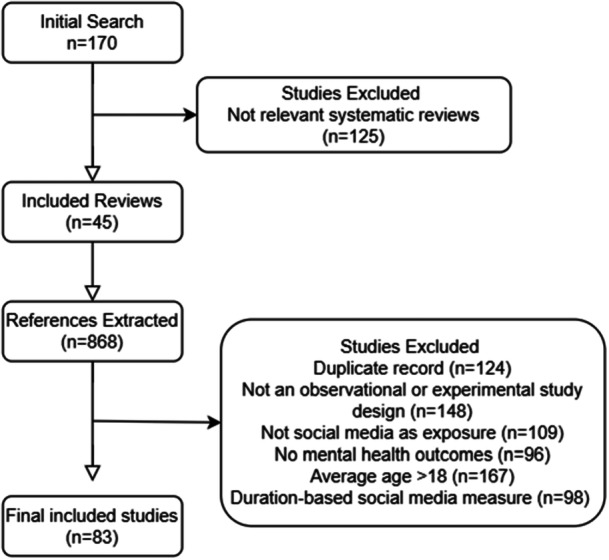
PRISMA 2020 flow diagram for scoping reviews.

The primary articles extracted from the systematic reviews were uploaded to Rayyan, a platform for systematic review management and coding (Ouzzani et al. 2016). Duplicate records were removed, and the remaining titles and abstracts were screened by the primary reviewer (AS) to determine eligibility according to the inclusion and exclusion criteria. Eligible studies were fully reviewed and assessed by two reviewers (AS, MA). The study selection flow chart detailing the number of reviews and extracted primary articles that were included and excluded, including reasons for exclusion, are detailed in Figure 1.

### 2.2 Inclusion and Exclusion Criteria

Observational and experimental studies published in a peer‐reviewed journal were eligible for inclusion. Studies were included if they examined the relationship between non‐duration‐based social media use as the exposure, and one or more mental health outcomes in a youth population with an average age ≤18. The study was included if the average age of the study participants was ≤18. Both objective and self‐report measures of social media were included, but studies reporting on general internet usage or screen time where social media use was not disaggregated from other online activities were excluded, as were studies where the measure of social media use was solely duration‐based. Examples of studies that were excluded for this reason were those that solely used a measurement such as: “hours per day/month spent using social media”, “average time on social media per week” or “number of times checking social media in a day” to ascertain the exposure. For the outcome, our operational definition of mental health was intentionally broad, and included both positive indicators of well‐being (i.e. life satisfaction, self‐esteem) as well as negative ones (i.e. depression, anxiety, loneliness). Hereafter, we use the term ‘outcome’ to refer to the role of mental health as a dependent variable, not necessarily as a measure of clinical significance.

### 2.3 Data Extraction, Risk of Bias, and Quality Assessment

Two reviewers (AS, MA) extracted the following data for all studies meeting inclusion criteria: study design (e.g. cross‐sectional), years of data collection, study sample country and age range, descriptions of SMU measures, mental health outcomes, effect sizes, and measures of uncertainty (where available). In studies with multiple SMU measures and/or mental health outcomes, each association was extracted separately.

Non‐randomized studies included in this review were critically assessed, using an adapted version of the Newcastle‐Ottawa quality assessment scale (NOS) (Wells et al. 2000). The NOS quantifies the quality of an observational study by considering the study selection, the comparability of the groups, and the assessment of the exposures and outcomes of interest. Studies were assigned a “star rating” on a 10‐point scale, based on criteria specific to this study (see Appendix 3). Studies were considered high quality if they received 8–10 stars, moderate from 5 to 7 stars, and low from 0 to 4 stars.

## 3 Results

### 3.1 Study Selection

After removing duplicates, 868 unique studies were extracted from 45 systematic reviews. After title and abstract review, 126 studies were selected for full‐text review, of which 84 studies met the final inclusion criteria. A flow chart detailing the study selection process with reasons for study exclusion is presented in Figure 1.

### 3.2 Study Characteristics

The summary study characteristics are presented in Table 1, and in‐detail in Supplementary Table A. A total of 84 studies assessed the relationship between non‐duration‐based social media use and adolescent mental health, and 163 separate estimates were reported reflecting the multiple associations tested in some studies. Studies were published between 2006 and 2023, using data collected from 2008 to 2022 (*n* = 20 studies did not report the date of data collection). Most studies utilized a cross‐sectional study design (*n* = 61), often with a convenience sample of participants (*n* = 70). Sample sizes ranged from 41 to 154,981 (Mean = 5,760; median = 598.5; IQR = 833.5; SD = 23,488). The studies were conducted across 36 countries, with samples being most frequently from the United States (*n* = 14), China (*n* = 8), and Australia (*n* = 7). Almost all studies recruited both boys and girls (*n* = 78), though six focused on solely female samples. Population ages ranged from 10 to 23 years; six studies did not report on age, rather using school grade to define their population of interest. The majority of studies (*n* = 59) did not focus on a specific platform; among those that did, Facebook (*n* = 18), Instagram (*n* = 2), and Twitter (*n* = 1) platforms were selected.

**Table 1:** Characteristics of included studies.

	*N*/mean	%/SD
Study design		
Cross‐sectional	61	73.5%
Cohort	22	26.5%
Region		
North America	16	19.3%
Europe	33	39.8%
Asia	23	27.7%
Australia	7	8.4%
Africa	2	2.4%
Global	2	2.4%
**Years of data collection**	2008–2022	
Sex		
Female only	6	7.2%
All	77	92.7%
**Age**	10–23	
Platform		
Specific	59	71.1%
Non‐specific	24	28.9%
SMU domains*		
SMU Measures	41	49.4%
Addiction‐like measures	30	36.1%
Structural aspects	16	19.3%
Interactions on SM	7	8.4%
Feelings about SM	21	25.3%
Mental health outcome*		
Depression	40	48.2%
Anxiety	19	22.9%
Psychological well‐being	13	15.7%
ADHD	4	4.8%
ODD	1	1.2%
Suicidal ideation	4	4.8%
Self‐esteem	10	12.0%
General psychopathology	9	10.8%
Body image dissatisfaction/eating disorders	21	25.3%

Abbreviations: ADHD, Attention‐Deficit/Hyperactivity Disorder; ODD, Oppositional Defiant Disorder; SM, social media; SMU, social media use.

* Percentages sum to greater than 100% as studies often reported on more than one mental health outcome.

### 3.3 Summary of Social Media Use Measures

Studies included 85 distinct SMU measures, outlined in Supplementary Table B. In order to summarize across studies, these measures were grouped into five domains based on their shared characteristics and key features. The first domain, “SMU Habits” included measures that assess specific aspects of adolescents’ social media activities, such as their habits related to posting, commenting, or editing photos. This domain included validated scales that aggregated several of these use measures into a single tool, such as the Use of Facebook Questionnaire (Errasti et al. 2017) or the Social Networking Activity Intensity Scale (SNAIS) (Li et al. 2016). The second domain, “Addiction‐like Measures” included tools designed to assess problematic social media use or addiction, such as the Bergen Social Media Addiction Scale (BSMAS) (Andreassen et al. 2012) or the Social Media Disorder Scale (van den Eijnden et al. 2016). This domain also included single items assessing components of these scales, such as ‘conflicts with daily life’. The third domain, “Structural Aspects” included measures that focus on features of users’ social media profiles themselves, such as the number of friends/followers, or number of platforms used. The fourth domain, “Interactions” included measures designed to capture adolescents’ interactions with others on social media. This domain covered aspects such as social media‐based social interaction, establishing relationships, and negative online experiences, such as unwanted contact, racism, or bullying. The fifth domain “Feelings about Social Media” included measures that assessed internal processes related to social media use, such as self‐presentation, emotional investment, and social media fatigue.

### 3.4 Mental Health Outcomes

Mental health outcomes and their associated measures are presented in detail in Supplementary Table C. Nearly half of all studies included a measure of depression or depressive symptoms (*n* = 41) (Li et al. 2016; Banjanin et al. 2015; Bányai et al. 2017; Barry et al. 2017; Blomfield neira and Barber 2014; Bonsaksen et al. 2022; Charmaraman et al. 2021; Coyne et al. 2023a; Dhir et al. 2018; Fardouly et al. 2020; Frison and Eggermont 2015; Ghergut and Maftei 2021; Akkın Gürbüz et al. 2017; Hanprathet et al. 2015; Khalil et al. 2022; Li et al. 2017; Morin‐Major et al. 2016; Muzi et al. 2021; Nesi et al. 2021a; Niu et al. 2018; The digital footprints of adolescent depression 2019; Pontes 2017; Skogen et al. 2021; 2023; Tao and Fisher 2022; Thomas et al. 2023; Tian et al. 2018; Wang et al. 2018; Watson et al. 2022; Woods and Scott 2016; Xie et al. 2018; Yurdagül et al. 2021; Boer et al. 2021; Cheng et al. 2023; Fredrick et al. 2022; Gingras et al. 2023; Nesi et al. 2022; Puukko et al. 2020; Raudsepp and Kais 2019; Vernon et al. 2017). Anxiety and anxiety symptoms were measured in nineteen studies (Barry et al. 2017; Dhir et al. 2018; Fardouly et al. 2020; Ghergut and Maftei 2021; Hanprathet et al. 2015; Khalil et al. 2022; Muzi et al. 2021; Pontes 2017; Skogen et al. 2023, 2024; Tao and Fisher 2022; Thomas et al. 2023; Woods and Scott 2016; Yurdagül et al. 2021; Gingras et al. 2023; Azhari et al. 2022; Caner et al. 2022; Kim et al. 2020; Louragli et al. 2019). Six of these studies focused on social anxiety specifically (Charmaraman et al. 2021; Dhir et al. 2018; Fardouly et al. 2020; Khalil et al. 2022; Yurdagül et al. 2021; Caner et al. 2022). Thirteen studies used an assessment of general psychopathology, such as psychological difficulties, internalizing symptoms, or poor mental health as the outcome of interest (Coyne et al. 2023a; Hanprathet et al. 2015; Muzi et al. 2021; Wang et al. 2018; Cheng et al. 2023; Apaolaza et al. 2014; Boer et al. 2020a; Marino et al. 2020; Morello et al. 2024; Nesi et al. 2021b; Walsh et al. 2020; Chen et al. 2021; Dumas et al. 2023). Attention problems/Attention Deficit Hyperactivity Disorder (ADHD) (Barry et al. 2017; Muzi et al. 2021; Nesi et al. 2021b; Boer et al. 2020b) and suicidal behavior (Khalil et al. 2022; Nesi et al. 2021b; Swedo et al. 2021; Dunlop et al. 2011) were assessed in four studies each, and Oppositional Defiant Disorder (ODD) was assessed in just one (Barry et al. 2017).

Studies used a wide range of scales to assess mental health outcomes (*n* = 90), and all but three had published psychometric evidence supporting their reliability and validity. Among the 41 studies assessing depression, 17 different scales were used (e.g., CES‐D, PHQ, CDI), with no single instrument applied in more than one‐third of studies. Anxiety was assessed using 17 distinct scales across 19 studies, including both general measures (e.g., GAD‐7) and disorder‐specific instruments (e.g., Social Anxiety Inventory). Across all outcomes, few studies used the same mental health measure, underscoring the lack of standardization in outcome assessment. Additionally, studies operationalized mental health outcomes using broad psychosocial constructs. Ten studies examined the impact that social media has on self‐esteem, social self‐esteem, or self‐concept (Errasti et al. 2017; Bányai et al. 2017; Blomfield neira and Barber 2014; Morin‐Major et al. 2016; Niu et al. 2018; Wang et al. 2018; Woods and Scott 2016; Valkenburg et al. 2006; Metzler and Scheithauer 2017; Valkenburg et al. 2017). Loneliness, FOMO (i.e., fear of missing out), and stress were examined in nine studies (Barry et al. 2017; Morin‐Major et al. 2016; Pontes 2017; Tian et al. 2018; Nesi et al. 2022; Azhari et al. 2022; Tomczyk and Selmanagic‐Lizde 2018; Teppers et al. 2014; Wang et al)Positive measures of mental health such as wellbeing and life satisfaction were assessed in thirteen studies (Muzi et al. 2021; Skogen et al. 2023, 2024; Tian et al. 2018; Boer et al. 2022, 2021; Walsh et al. 2020; Valkenburg et al. 2006; Buda et al. 2021; Wenninger et al. 2014; Ziv and Kiasi 2016; Frison and Eggermont 2016; van den Eijnden et al. 2018). Twenty‐one studies examined the relationship between use of social media and adolescent body image, body dysmorphia, and eating behaviors (Coyne et al. 2023a; Fardouly et al. 2020; Ghergut and Maftei 2021; Muzi et al. 2021; Nesi et al. 2021a; Yurdagül et al. 2021; Valkenburg et al. 2006; Chang et al. 2019; de Vries et al. 2019; Ho et al. 2016; Hosokawa et al. 2023; Lonergan et al. 2020; Maftei 2022; McLean et al. 2015; Meier and Gray 2014; Nolan et al. 2023; Tadena et al. 2020; Wilksch et al. 2020; Yurtdaş Depboylu et al. 2022; Tiggemann and Slater 2017; Vandenbosch and Eggermont 2016).

### 3.5 Associations between Social Media Use and Mental Health

**Figure 2:**
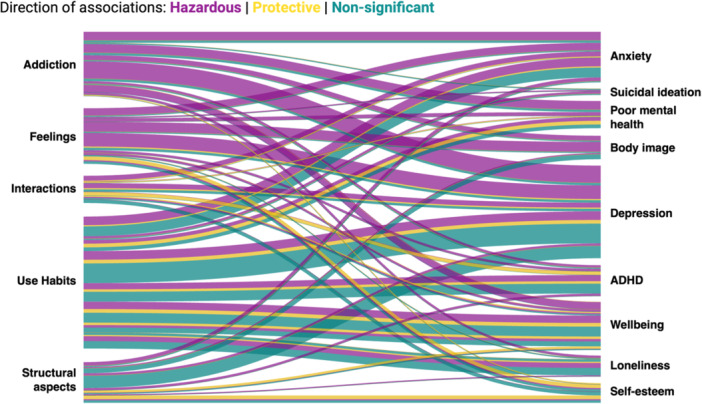
Associations between social media use measures and mental health outcomes. Purple pathways represent harmful associations, yellow pathways represent protective associations, and teal pathways represent non‐significant associations. The thickness of the pathway is representative of the number of studies reporting the association. ADHD, Attention Deficit Hyperactivity Disorder; MH, Mental Health; SI, Suicidal Ideation.

Associations between SMU measures and mental health outcomes are described in Table 2. For each outcome, social media measures are grouped according to the consistency of reported associations, including consistently harmful, consistently protective, no (statistically significant) association, inconsistent, and insufficient findings. Results were categorized as *inconsistent* if there was no clear trend across all associations and *insufficient* if they were reported in only one study estimate. Associations are also presented visually in Figure 2. Findings for each mental health outcome are described below.

**Table 2:** Summary of associations between social media measures and mental health outcomes.

Study measure	Direction of associated risk with mental health outcome			
	Harmful	Protective	Null	Overall
**Depression** b(*n* = 55)	**28**	**4**	**23**	
*SMU Habits*	*3*	*3*	*10*	
General use	2 (Ghergut and Maftei 2021; Thomas et al. 2023)	1 (Tian et al. 2018)	3 (Blomfield neira and Barber 2014; Niu et al. 2018; Boer et al. 2021)	↕
Passive use	1 (Cheng et al. 2023)		1 (Coyne et al. 2023a)	↕
Active use		1 (girls only) (Fredrick et al. 2022)	2 (Coyne et al. 2023a; Puukko et al. 2020)	∅
Posting behavior		1 (boys only) (Nesi et al. 2021a)	4 (Fardouly et al. 2020; Akkın Gürbüz et al. 2017; Morin‐Major et al. 2016; Nesi et al. 2021a)	∅
*Addiction‐like measures*	*12*		*2*	
Problematic/addictive use	12 (Bányai et al. 2017; Coyne et al. 2023a; Hanprathet et al. 2015; Li et al. 2017; Muzi et al. 2021; Pontes 2017; Wang et al. 2018; Watson et al. 2022; Yurdagül et al. 2021; Boer et al. 2021; Raudsepp and Kais 2019; Vernon et al. 2017)		2 (Khalil et al. 2022; Gingras et al. 2023)	↑
*Structural Aspects*	*2*		*10*	
Number of accounts	2 (Barry et al. 2017; Thomas et al. 2023)			↑
Number of friends/followers			4 (Banjanin et al. 2015; Morin‐Major et al. 2016; Nesi et al. 2021a; The digital footprints of adolescent depression 2019)	∅
Number of posts			3 (Banjanin et al. 2015; Nesi et al. 2021a; The digital footprints of adolescent depression 2019)	∅
Number of ‘likes’ received			3 (Fardouly et al. 2020; Nesi et al. 2021a; The digital footprints of adolescent depression 2019)	∅
*Interactions*	*3*		*1*	
Vicarious racial discrimination	2 (Tao and Fisher 2022; Thomas et al. 2023)		1 (Thomas et al. 2023)	↑
Individual racial discrimination	1 (Tao and Fisher 2022)			↕
*Feelings about social media*	*8*	*1*		
Investment in use	2 (Blomfield neira and Barber 2014; Woods and Scott 2016)			↑
Social comparison/appearance investment	5 (Charmaraman et al. 2021; Coyne et al. 2023a; Fardouly et al. 2020; Nesi et al. 2021a; Niu et al. 2018)			↑
Self‐presentation	1 (Skogen et al. 2021)	1 (Xie et al. 2018)		↕
**Anxiety** b (*n* = 26)	**21**	**2**	**3**	
*SMU Habits*				
General use	2 (Ghergut and Maftei 2021; Thomas et al. 2023)	1 (Maftei 2022)		↕
Following influencers/celebrities	2 (Charmaraman et al. 2021; Caner et al. 2022)			↑
*Addiction‐like measures*				
Problematic/addictive use	7 (Muzi et al. 2021; Pontes 2017; Yurdagül et al. 2021; Gingras et al. 2023; Caner et al. 2022; Kim et al. 2020; Louragli et al. 2019)		2 (Hanprathet et al. 2015; Khalil et al. 2022)	↑
*Structural aspects*				
Number of accounts used	3 (Barry et al. 2017; Thomas et al. 2023; Caner et al. 2022)			↑
*Interactions*				
Vicarious racism	2 (Tao and Fisher 2022; Thomas et al. 2023)			↑
Individual racial discrimination	1 (Thomas et al. 2023)	1 (Tao and Fisher 2022)		↕
*Feelings about social media*				
Social media fatigue	2 (Dhir et al. 2018; Kim et al. 2020)			↑
Social comparison/appearance investment	2 (Charmaraman et al. 2021; Fardouly et al. 2020)		1 (Fardouly et al. 2020)	↕
**General Psychopathology** b ^,^ c (*n* = 10)	**8**	**0**	**2**	
*SMU Habits*				
Passive use	1 (Cheng et al. 2023)		1 (Coyne et al. 2023a)	↕
*Addiction‐like measures*				
Problematic/addictive use	7 (Coyne et al. 2023a; Hanprathet et al. 2015; Watson et al. 2022; Boer et al. 2020a; Marino et al. 2020; Walsh et al. 2020; Chen et al. 2021)		1 (Muzi et al. 2021)	↑
**ADHD Symptoms** b ^,^ c ^,^ d (*n* = 2)	**2**	**0**	**0**	
*Addiction‐like measures*				
Problematic/addictive use	2 (Muzi et al. 2021; Boer et al. 2020b)			↑
**Suicidal Ideation/Behavior** a ^,^ b ^,^ c (*n* = 2)	**1**	0	**1**	
*SMU Habits*				
Posting/engaging with suicide‐related information	1 (Swedo et al. 2021)		1 (Dunlop et al. 2011)	↕
**Self‐Esteem/Self‐Concept** (*n* = 18)	**5**	**4**	**9**	
*SMU Habits*				
General use	1 (Blomfield neira and Barber 2014; Niu et al. 2018)	1 (Blomfield neira and Barber 2014; Niu et al. 2018)	2 (Blomfield neira and Barber 2014; Valkenburg et al. 2017)	↕
Posting on Facebook	1 (Errasti et al. 2017)		1 (Morin‐Major et al. 2016)	↕
*Structural aspects*				
Number of friends		1 (Metzler and Scheithauer 2017)	2 (Errasti et al. 2017; Morin‐Major et al. 2016)	↕
Reactions (i.e., ‘likes’)	1 (Metzler and Scheithauer 2017)	2 (Valkenburg et al. 2023, 2019)		↕
*Interactions*				
Interacting/relationship building on SM			3 (Morin‐Major et al. 2016; Valkenburg et al. 2006; Metzler and Scheithauer 2017)	∅
*Feelings about social media*				
Investment in use	2 (Blomfield neira and Barber 2014; Woods and Scott 2016)	(Blomfield neira and Barber (2014); Woods and Scott (2016))	1 (Blomfield neira and Barber 2014)	↕
**Loneliness** (*n* = 13)	**3**	**5**	**5**	
*SMU Habits*				
General use	1(Pontes et al. 2017)	1 (Tian et al. 2018)	1 (Tomczyk and Selmanagic‐Lizde 2018)	↕
Posting on Facebook			2 (Morin‐Major et al. 2016; Azhari et al. 2022)	∅
Entertainment		1 (Morello et al. 2024)	1 (Teppers et al. 2014)	↕
*Addiction‐like measures*				
Problematic/addictive use	2 (Pontes 2017; Tomczyk and Selmanagic‐Lizde 2018)		1(Yurdagül et al. 2021)	↕
*Interactions*				
Use for socialization/interaction		3 (Morin‐Major et al. 2016; Morello et al. 2024; Teppers et al. 2014)		↓
**Wellbeing/Life Satisfaction** (*n* = 16)	**8**	**3**	**5**	
*SMU Habits*				
General use		1 (Ziv and Kiasi 2016)	3 (Tian et al. 2018; Boer et al. 2022, 2021)	↕
Passive use	2 (Wenninger et al. 2014; Frison and Eggermont 2016)			↑
*Addiction‐like measures*				
Problematic/addictive use	6 (Boer et al. 2022, 2021; Walsh et al. 2020; Buda et al. 2021; van den Eijnden et al. 2018; Naeemi and Tamam 2017)			↑
*Structural Aspects*				
Number of friends/followers		1 (Tiggemann and Slater 2017)	1 (Wenninger et al. 2014)	↕
*Interactions*				
SMU for relationship building/interacting		1 (Wenninger et al. 2014)	1 (Valkenburg et al. 2006)	↕
**Eating Disorders/Body Image Dissatisfaction** b (*n* = 23)	**14**	**2**	**7**	
*SMU Habits*				
General use	2 (Ghergut and Maftei 2021; de Vries et al. 2019)			↑
Posting behavior		2 (Nesi et al. 2021a; Chang et al. 2019)	2 (Fardouly et al. 2020; McLean et al. 2015)	↕
Editing selfies	1 (Lonergan et al. 2020)		1 (Chang et al. 2019; McLean et al. 2015)	↕
*Addiction‐like measures*				
Problematic/addictive use	4 (Muzi et al. 2021; Yurdagül et al. 2021; Nolan et al. 2023; Yurtdaş Depboylu et al. 2022)		1 (Coyne et al. 2023a)	↑
*Structural aspects*				
Number of ‘likes’ on posts			2 (Fardouly et al. 2020; Nesi et al. 2021a)	∅
*Feelings about social media*				
Social comparison	4 (Coyne et al. 2023a; Fardouly et al. 2020; Ho et al. 2016; Vandenbosch and Eggermont 2016)			↑
Appearance/photo investment	3 (Fardouly et al. 2020; Lonergan et al. 2020; McLean et al. 2015)		1 (Nesi et al. 2021a)	↑

*Notes:* Only measures examined in more than one study are included. Social media (SM), Social media use (SMU); ↑ = increased risk; ↓ = decreased risk, ∅ = non‐significant associations, ↕ = inconsistent associations

^a^ no positive associations were reported;

^b^ no negative associations were reported;

^c^ no inconsistent associations were reported;

^d^ no null associations were reported; consistency of findings.

### 3.6 Depression

Harmful associations were reported between five SMU measures and depressive symptoms. First, problematic or addictive use of social media was associated with greater depressive symptoms in 13 studies (Bányai et al. 2017; Bonsaksen et al. 2022; Coyne et al. 2023a; Hanprathet et al. 2015; Li et al. 2017; Muzi et al. 2021; Pontes 2017; Wang et al. 2018; Watson et al. 2022; Yurdagül et al. 2021; Boer et al. 2021; Raudsepp and Kais 2019; Vernon et al. 2017), and a null association was reported in two studies (Khalil et al. 2022; Gingras et al. 2023). Second, measures of social comparison and appearance investment were associated with depressive symptoms in five studies (Charmaraman et al. 2021; Coyne et al. 2023a; Fardouly et al. 2020; Nesi et al. 2021a; Niu et al. 2018). Lastly, the number of accounts an adolescent reported using (Barry et al. 2017; Thomas et al. 2023), vicarious racial discrimination (Tao and Fisher 2022; Thomas et al. 2023), and social media investment (Blomfield neira and Barber 2014; Woods and Scott 2016) were associated with depressive symptoms in two studies respectively.

No associations were found between the number of friends/followers, posts or likes on an individual's profile and depressive symptoms (Banjanin et al. 2015; Fardouly et al. 2020; Morin‐Major et al. 2016; Nesi et al. 2021a; The digital footprints of adolescent depression 2019). Similarly, studies that measured active use (e.g., posting, liking and commenting) (Coyne et al. 2023a; Fredrick et al. 2022; Puukko et al. 2020), or posting alone (Fardouly et al. 2020; Akkın Gürbüz et al. 2017; Morin‐Major et al. 2016; Nesi et al. 2021a) found no associations with depressive symptoms.

Inconsistent findings were reported for four social media measures. First, studies of general social media usage (e.g. aggregate scales of use measures) found harmful (Ghergut and Maftei 2021; Thomas et al. 2023), protective (Tian et al. 2018), and null (Blomfield neira and Barber 2014; Niu et al. 2018; Boer et al. 2021) associations with depressive symptoms. Second, measures of passive social media use (e.g. browsing or scrolling) found both harmful (Cheng et al. 2023) and null associations (Coyne et al. 2023a). Third, associations from experiences of individual racial discrimination were harmful (Tao and Fisher 2022), and null (Thomas et al. 2023). Lastly, studies of self‐presentation also found both harmful (Skogen et al. 2021) and protective (Xie et al. 2018) associations with depression.

There was insufficient evidence for associations between depression and 19 SMU measures as they were examined in only 1 study each. These included general social media use (Li et al. 2021), following celebrities (Charmaraman et al. 2021), curating your feed (Coyne et al. 2023a), negative experiences on social media (Skogen et al. 2023), social media fatigue (Dhir et al. 2018), social media envy (Cheng et al. 2023), positive emotional responses to social media (Nesi et al. 2022), perceived social support on social media (Frison and Eggermont 2015), interacting on social media (Morin‐Major et al. 2016), looking at a friend's profile (Akkın Gürbüz et al. 2017), video sharing (Akkın Gürbüz et al. 2017), civic engagement (Tao and Fisher 2022), mindful media use (Coyne et al. 2023a), parental monitoring of social media use (Barry et al. 2017), peer feedback concern (Nesi et al. 2021a), and negative emotional responses to social media (Nesi et al. 2022).

Overall, depression symptom increases were consistently associated with problematic or addictive social media use (i.e., 12/14 reported associations were significant) and social comparison (100% of associations). Protective effects of social media use on depressive symptoms were limited.

### 3.7 Anxiety

Five measures demonstrated a harmful association with anxiety symptoms across multiple studies. First, problematic or addictive use of social media was associated with greater anxiety symptoms in seven studies (Muzi et al. 2021; Pontes 2017; Yurdagül et al. 2021; Gingras et al. 2023; Caner et al. 2022; Kim et al. 2020; Louragli et al. 2019), and a null association was reported in two studies (Hanprathet et al. 2015; Khalil et al. 2022). Second, the number of accounts an adolescent reported using was associated with anxiety symptoms in three studies (Barry et al. 2017; Thomas et al. 2023; Caner et al. 2022). Lastly, social media fatigue (Dhir et al. 2018; Kim et al. 2020), following influencers/celebrities (Charmaraman et al. 2021; Caner et al. 2022), and vicarious racial discrimination (Tao and Fisher 2022; Thomas et al. 2023) were associated with increased anxiety symptoms in two studies each. No protective or null associations between SMU and anxiety were reported.

Inconclusive findings were reported for three types of social media use measures. First, studies of general social media use reported harmful (Ghergut and Maftei 2021; Thomas et al. 2023), and protective (Maftei 2022) associations with anxiety symptoms. Second, studies of individual racial discrimination on social media reported harmful (Thomas et al. 2023) and protective (Tao and Fisher 2022) associations. Third, studies of social comparison and appearance investment reported harmful (Charmaraman et al. 2021; Fardouly et al. 2020) and null (Fardouly et al. 2020) associations.

There was insufficient evidence for 15 measures as they were examined in only 1 study each. These included following nutrition and gaming influencers (Caner et al. 2022), negative experiences on social media (Skogen et al. 2023), social media investment (Woods and Scott 2016), self‐presentation on social media (Skogen et al. 2021), posting on social media (Fardouly et al. 2020; Azhari et al. 2022), following make‐up or funny influencers (Caner et al. 2022), parental monitoring of social media use (Barry et al. 2017), number of likes received (Fardouly et al. 2020), or civic engagement (Tao and Fisher 2022).

Overall, problematic/addictive use of social media, and the number of social media accounts were the two measures most consistently associated with increased anxiety, observed across 78% and 100% of associations respectively. The remainder of associations were generally inconclusive, or lacked sufficient evidence to draw conclusions.

### 3.8 General Psychopathology

Only problematic or addictive use of social media was found to be consistently associated with greater psychopathology, with harmful associations observed across seven (Coyne et al. 2023a; Hanprathet et al. 2015; Watson et al. 2022; Boer et al. 2020a; Marino et al. 2020; Walsh et al. 2020; Chen et al. 2021) of eight (Hosokawa et al. 2023) studies. No studies reported consistently protective or null associations.

Inconclusive findings were reported for studies of passive social media (e.g., browsing, scrolling), reporting harmful (Cheng et al. 2023) and null (Coyne et al. 2023a) associations with general psychopathology.

There was insufficient evidence for 11 measures as they were examined in only 1 study each. These included studies examining general social media use (Boer et al. 2020a), cleaning your feed (Coyne et al. 2023a), social comparison (Coyne et al. 2023a), social media envy (Nesi et al. 2021b), negative emotional responses to social media (Cheng et al. 2023), and socializing (Apaolaza et al. 2014).

Overall, problematic/addictive use of social media was most consistently associated with increased general psychopathology, with a harmful effect observed across 88% of observed associations.

### 3.9 ADHD Symptoms

There was an observed harmful association between problematic or addictive use of social media and ADHD symptoms in two studies (Muzi et al. 2021; Boer et al. 2020b). No studies reported consistently protective, null, or inconclusive associations.

There was insufficient evidence for an association between ADHD and four SMU measures. These included the number of accounts the adolescent reported using (Barry et al. 2017), general social media use (Boer et al. 2020b), parental monitoring of social media (Barry et al. 2017), and negative emotional responses to social media (Nesi et al. 2021b).

Overall, there was a lack of evidence to draw strong conclusions about the relationship between social media use and ADHD symptoms. Problematic/addictive use was associated with worse mental heath outcomes across two studies, and the remainder of associations were only examined in one study each.

### 3.10 ODD Symptoms

Only one study examined ODD symptoms, finding that the number of accounts the adolescent reported was associated with increased ODD symptoms, but that parental monitoring was not (Barry et al. 2017).

### 3.11 Suicidal Ideation/Behavior

There were no consistently harmful or protective associations reported between SMU measures and suicidality. Inconclusive findings were reported for one type of measure, posting or engaging with content related to suicide on social media. Studies using this measure reported harmful (Swedo et al. 2021), and null associations (Dunlop et al. 2011).

There was insufficient evidence for two measures, including seeing suicide‐related information on social media (Khalil et al. 2022), and negative emotional responses to social media (Nesi et al. 2021b).

### 3.12 Self‐Esteem/Self‐Concept

There were no consistently harmful or protective associations reported between SMU measures and self‐esteem. Inconclusive findings were reported for five types of social media use measures. First, studies of general social media use reported protective (Blomfield neira and Barber 2014), harmful (Niu et al. 2018), and null (Blomfield neira and Barber 2014; Valkenburg et al. 2017) associations with self‐esteem. Second, posting on Facebook demonstrated both harmful (Errasti et al. 2017) and null (Morin‐Major et al. 2016) associations. Third, studies measuring the number of friends or followers an adolescent had reported protective (Metzler and Scheithauer 2017) and null (Errasti et al. 2017; Morin‐Major et al. 2016) associations. Lastly, studies looking at profile reactions (e.g., likes) found both protective (Valkenburg et al. 2023, 2019) and harmful (Metzler and Scheithauer 2017) associations with self‐esteem.

There was insufficient evidence for eight measures. These included problematic/addictive use of social media (Bányai et al. 2017), posting on twitter (Errasti et al. 2017), expressing emotions on twitter (Errasti et al. 2017), social comparison (Niu et al. 2018), expressing emotions or empathy on Facebook (Errasti et al. 2017), and self‐presentation on social media (Metzler and Scheithauer 2017).

In summary, the evidence for the relationship between social media use and self‐esteem is largely inconclusive. Of the 18 associations examined, 5 were harmful, 4 were protective, and 9 were null.

### 3.13 Loneliness

There were no consistently harmful associations between SMU and loneliness. There was a protective association between interacting on social media (e.g. making friends, chatting) and loneliness, which was reported in three studies (Morin‐Major et al. 2016; Morello et al. 2024; Teppers et al. 2014). There was no association between posting on social media and loneliness (Morin‐Major et al. 2016; Azhari et al. 2022).

Inconclusive findings were reported for three types of social media use measures. First, studies of general social media use reported harmful (Wang et al) protective (Tian et al. 2018), and null (Tomczyk and Selmanagic‐Lizde 2018) associations with loneliness. Second, studies of social media use for entertainment reported protective (Morello et al. 2024) and null (Teppers et al. 2014) associations. Third, studies of problematic or addictive use of social media reported harmful (Pontes 2017; Tomczyk and Selmanagic‐Lizde 2018) and null (Yurdagül et al. 2021) associations with loneliness.

There was insufficient evidence for the association between nine measures and loneliness. These included social media use for coping (Morello et al. 2024), conformity (Morello et al. 2024), social skills compensation (Teppers et al. 2014), decreasing loneliness (Teppers et al. 2014), the number of accounts used (Barry et al. 2017), the number of friends/followers (Morin‐Major et al. 2016), positive and negative emotional responses (Nesi et al. 2022), and parental monitoring of social media (Barry et al. 2017).

Overall, loneliness represented one of the few domains where there was a consistently protective association observed between SMU and mental health. Specifically, making friends on social media was associated with decreased loneliness across three studies.

### 3.14 Wellbeing

A harmful association was found between two SMU measures and wellbeing. First, problematic/addictive use of social media was associated with decreased wellbeing in six studies (Boer et al. 2022, 2021; Walsh et al. 2020; Buda et al. 2021; van den Eijnden et al. 2018; Naeemi and Tamam 2017). Second, passive social media use such as browsing or scrolling was found to be harmful in two studies (Wenninger et al. 2014; Frison and Eggermont 2016). No consistently protective or null associations were observed.

Inconclusive findings were reported for three types of social media use measures. First, studies of general social media use reported both protective (Ziv and Kiasi 2016), and null (Tian et al. 2018; Boer et al. 2022, 2021) associations with wellbeing. Second, studies of the number of friends/followers reported protective (Tiggemann and Slater 2017) and null (Wenninger et al. 2014) associations. Third, studies of interacting on social media reported both protective (Wenninger et al. 2014) and null (Valkenburg et al. 2006) associations with wellbeing.

There was insufficient evidence for six measures, including posting on social media (Wenninger et al. 2014), the number profile reactions (e.g., likes, comments) (Valkenburg et al. 2006), negative experiences on social media (Skogen et al. 2023), self‐presentation (Skogen et al. 2021), social comparison (Frison and Eggermont 2016), and commenting on social media posts (Wenninger et al. 2014).

Overall, there is consistent evidence to suggest that problematic/addictive use of social media is associated with decreased wellbeing, with a harmful association observed in all studies where it was tested (*n* = 6).

### 3.15 Body Dissatisfaction Measures

There was harmful association observed between four types of social media measures and body dissatisfaction/eating disorders. First, problematic or addictive use of social media was associated with greater body concerns in four studies (Muzi et al. 2021; Yurdagül et al. 2021; Nolan et al. 2023; Yurtdaş Depboylu et al. 2022), and a null association was reported in one study (Coyne et al. 2023a; Hanprathet et al. 2015; Khalil et al. 2022). Second, social comparison was associated with body concerns in four studies (Coyne et al. 2023a; Fardouly et al. 2020; Ho et al. 2016; Vandenbosch and Eggermont 2016). Third, three studies on appearance and photo investment reported harmful associations (Fardouly et al. 2020; Lonergan et al. 2020; McLean et al. 2015), and one reported a null association (Nesi et al. 2021a). Lastly, general social media use showed a harmful association with body concerns in two studies (Ghergut and Maftei 2021; de Vries et al. 2019). We identified no consistently protective associations.

Two types of measures were not associated with body concerns such as body dissatisfaction or eating disorders. First, the number of likes received was not associated with body concerns in two studies (Fardouly et al. 2020; Nesi et al. 2021a). Second, passive social media use such as browsing or scrolling was not associated with body concerns in two studies (Coyne et al. 2023a; Chang et al. 2019).

Inconclusive findings were reported for two types of social media use measures. First, studies of posting on social media reported both protective (Nesi et al. 2021a; Chang et al. 2019), and null (Fardouly et al. 2020; McLean et al. 2015) associations with body concerns. Second, studies on photo manipulation reported both harmful (Lonergan et al. 2020) and protective (Chang et al. 2019; McLean et al. 2015) associations.

There was insufficient evidence for 13 measures. These included following celebrities/thinness related accounts (Hosokawa et al. 2023), avoidance of posting selfies (Lonergan et al. 2020), appearance exposure (Meier and Gray 2014), the number of social media accounts (Wilksch et al. 2020), social media affinity (Tadena et al. 2020), active social media use (Coyne et al. 2023a), body concerns, curating your feed (Coyne et al. 2023a), mindful media use (Coyne et al. 2023a), taking intentional breaks from social media (Coyne et al. 2023a), the number of friends or followers (Nesi et al. 2021a), and the number of selfies posted (Nesi et al. 2021a).

In summary, problematic/addictive use of social media was consistently associated with increased body concerns in adolescents across 80% of observed associations. Social comparison and appearance concern were also harmful, reported across 100% and 75% of associations respectively. Only two associations were protective, each reported in one study only, indicating there is little evidence to suggest that SMU is beneficial for body concerns in adolescent populations.

### 3.16 Study Quality

Study quality varied; around half of the studies (*n* = 44) were considered to be at moderate risk for bias according to the NOS assessment (Supplementary Table D). Notably, 35 studies received less than two stars for comparability, indicating that their analyses did not consider or adjust for the influence of confounding variables. Other predominant study weaknesses included a lack of justification for the study sample size, and an overreliance on convenience sampling.

## 4 Discussion

Nearly ubiquitous among youth today, social media has become a dynamic and complex part of adolescent social development, with features that may be associated with harmful, beneficial, and benign effects on mental health. The aim of this study was to review published research of social media and mental health outcomes in adolescents, in order to summarize the associations between specific SMU measures and mental health. We purposefully excluded studies that focused on time spent using social media. These duration‐based studies accounted for 61% of the literature that otherwise met our inclusion criteria. Across the included studies, we note four central findings. First, exposure to social media is measured with a wide array of questions, instruments, and analytic methods, and information on the validity of stated approaches was limited. Second, measures of social comparison and addiction‐related use were most consistently associated with poor mental health. Third, evidence that aspects of social media provided beneficial or protective associations with mental health varied substantially across mental health outcomes. Fourth, despite the substantial size of the literature to date, most research has focused on limited mental health outcomes and study populations, with implications for the conclusions that can be currently drawn about the impact of social media in youth.

Across 84 studies, 85 distinct measures of social media use were used, and fewer than half (*n* = 37; 45%) of those provided any evidence of validity. Further, 49% of all studies did not provide enough information (i.e. standard error or confidence intervals, typically needed to be able to estimate any weighted pooled estimates). Substantial heterogeneity was also present in how the mental health outcomes were assessed, with 90 distinct instruments utilized, and little overlap in measures between studies. An initial goal of this study was to describe the magnitude of associations between SMU measures and mental health outcomes through meta‐analytic summary estimates and meta‐regression. However, between‐study variation in estimands, missing measures of uncertainty (e.g., standard errors), and the use of non‐standardized and ad‐hoc measures for both SMU and mental health meant that a quantitative summary was infeasible.

Despite the challenges, two SMU dimensions were consistently associated with poor mental health: social comparison and addiction‐related measures. The public nature of feedback and engagement may increase users' negative self‐evaluations, upward social comparisons, and harmful coping strategies (Nesi and Prinstein 2015; Nesi et al. 2018). These responses may disrupt the development of a positive self‐identity and healthy peer relationships and interfere with mental health over time. Studies employed various measures of social media addiction or dependence, but the documented validity of such measures is limited. Meta‐analysis of prevalence estimates across the world show substantial heterogeneity and concentrated prevalence among adolescents and young adults. Depending on the scale cut‐off specified, pooled prevalence estimates range from 5% for ‘severe’ to 25% for more lenient cut‐offs for categorizing disorder (note that the meta‐analysis included adult samples) (Cheng et al. 2021). The validity of social media addiction‐like measures is generally supported through tests of convergent validity, with higher scores on scales correlating with other dimensions commonly associated with addiction disorders (Andreassen et al. 2017). Scales such as the Bergen Facebook Addiction Scale (Andreassen et al. 2012) measure canonical dependence phenotypes such as impaired control over use, urgency to use, unsuccessful quit attempts, and negative impacts in role function and sleep. However, critics of the addiction framework for social media use argue that many of these concepts in addiction are insufficiently characterized (e.g., tolerance and withdrawal), and that pathologizing patterns of SMU may be premature (Panova and Carbonell 2018). It is also important to consider that many items on addiction‐like SMU scales may conceptually represent a symptom manifestation of depression and anxiety. This overlap underscores the importance of carefully considering timing and study design, as cross‐sectional associations may reflect reverse causation (i.e., mental health symptoms leading to higher problematic SMU scores) rather than addictive use of SMU causing mental health problems. Further, social media addiction literature is rapidly growing, and is outpacing meaningful connections with relevant theoretical addiction frameworks (Sun and Zhang 2021). This connection is necessary to do meaningful research that is responsive to the rapidly changing landscape of social media platforms and use patterns.

Overall, there was limited evidence reporting beneficial associations between social media and mental health. In fact, a consistently protective association only emerged for one measure; three studies reported decreased loneliness associated with use of social media for socialization/interaction. Individual studies reported protective associations for certain SMU measures, including active SMU, use of social media for entertainment, self‐presentation online, etc., suggesting potential use behaviors for further study. Indeed, certain aspects of social media may conceptually facilitate positive mental health. Anonymous use allows the user to control what personal information is shared, in content, format (e.g., text, audio, visual), and timing, which may increase opportunities for approval and social acceptance in turn. Self‐disclosure is an important part of adolescent development and has been shown to increase help‐seeking behavior (Valkenburg and Peter 2011), and online anonymity may particularly benefit those who are uncomfortable self‐disclosing. Posting or sharing this type of content, particularly self‐generated content, may be effective in regulating self‐harm urges or feelings of distress, or as a way to find community with shared experiences (Dyson et al. 2016). Online communities may provide opportunities to give and receive social support, improving health outcomes (Wang et al. 2011, 2006). Finally, social media may increase access to mental health promoting resources, by exposing users to meaningful conversations, normalizing help‐seeking, providing informational resources, and reducing the stigma around mental health (Betton et al. 2015; O'Reilly et al. 2018). This source of information and support can be used to engage individuals in treatment for mental health problems or as an avenue to deliver preventive interventions for at‐risk populations (Robinson et al. 2016). Adolescents report frequently seeking mental health resources on social media (O'Reilly et al. 2019).

Despite the large research base, very few studies focused on mental health conditions beyond depression and anxiety, especially those which have exhibited similar increasing trends in youth, such as suicidal ideation (Xiao et al. 2021) and ADHD (Safer 2018). Social media platforms often prioritize content that is high in arousal and delivered rapidly, which may decrease users' ability to engage in tasks requiring sustained attention (Thorell et al. 2022) and to regulate their attention internally after having gotten used to external sources (Madore et al. 2020). More research is needed to understand the role of SMU in increasing attentional challenges and other risks in youth.

In addition, more research is also needed to examine heterogeneity of effects across socio‐demographic groups, including race, ethnicity, gender identity, sexual orientation, and rurality. Social media experiences may be highly racialized (Zhang and Leung 2015; Wang et al. 2018) due to differences in social practices and exposure to traumatizing content, including interpersonal discrimination, racist images, and instances of violence toward people of color (Tao and Fisher 2022; Portillo et al. 2022; Del Toro and Wang 2023; Tynes et al. 2020). Studies examining social media use among sexual and gender minority (SGM) groups have reported protective mental health effects (Coyne et al. 2023b) due to increased opportunities for social support and identity exploration (Berger et al. 2022; Escobar‐Viera et al. 2018). However, SGM youth are also more likely to experience cyberbullying (Escobar‐Viera et al. 2018; Cénat et al. 2015), increasing their risk of psychological distress, depression, and suicidality (Escobar‐Viera et al. 2018; Cénat et al. 2015; Duong and Bradshaw 2014). To our knowledge, no study has examined social media use and mental health comparing adolescents living in urban versus rural communities, though studies in rural populations have reported both positive and negative effects (Nie et al. 2023; Anderson 2019). Self‐disclosure and access to mental health resources may be especially beneficial for those who lack sources of formal mental‐health support, including for residents of rural communities. Youth in rural areas often face significant challenges in accessing supportive mental health services (Robinson et al. 2017), as well as greater stigma (van Vulpen et al. 2018).

This study has limitations worth noting. Our search strategy was global in scope but limited to English language publications. This approach excluded not only reports written in other languages, but also investigations of social media platforms that are not in English. Major global platforms such as Weixin (WeChat) and Sina Weibo in China, VKontakte (VK) in Russia, and KakaoTalk in South Korea host hundreds of millions of users, each with unique experiences and potential mental health implications. Additionally, the classification of SMU behaviors and mental health outcomes reflect the categories and labels used in studies included in this review, as well as the judgment of co‐authors. We acknowledge that alternative classifications and interpretations are plausible, and accordingly, we have presented study‐level results to ensure transparency in our conclusions. Finally, we did not include outcomes related to risky alcohol and other substance use. These behaviors are comorbid with many of the included mental health outcomes and may be related to social media use in unique ways.

The impact of social media on young people's mental health has been identified as a public health emergency by researchers, policymakers, school administrators, and other stakeholders. While we agree with the US Surgeon General that ‘in an emergency, you don't have the luxury to wait for perfect information’ (Corredor‐Waldron et al. 2024), our hope is that this review will highlight specific and modifiable social media features and behaviors to target in order to create a healthier social media environment for youth. At the same time, addressing key research gaps we have identified will strengthen the effectiveness of existing interventions and increase future efforts to reduce growing adolescent mental health morbidity. Social media has become a central component of adolescent development. Helping youth navigate adolescence successfully will require minimizing the mental health hazards from its use.

## Funding

The authors received no specific funding for this work.

### 1 Ethics Statement

The authors have nothing to report.

## Conflicts of Interest

The authors declare no conflicts of interest.

## Supporting information

Supplementary Files.

## Data Availability

The data that support the findings of this study are available from the corresponding author upon reasonable request.
