# Supplementary material for: Time Out: A Scoping Review of Non‐Duration Based Social Media Use Measures and Adolescent Mental Health
Source: J Adolesc. 2025 Dec 19;98(3):659–77. doi: 10.1002/jad.70088 (PMC13044871; doi:10.1002/jad.70088)
Supplement: Supplementary file 1 — Supplementary Files. [file JAD-98-659-s001.docx]

Supplementary Table A: Characteristics of included studies

| Author | Country | Sample Size | Data Years | Social Media Platform | Age Range (Mean) |
| --- | --- | --- | --- | --- | --- |
| **Cross Sectional** | | | | | |
| Apaolaza, 2014 | China | 220 | 2013 | QZone | 14 – 19 (16.71) |
| Azhari, 2022 | UK | 41 | 2016 – 2017 | Any | 16-19 (17.83) |
| Banjanin, 2014 | Serbia | 336 | 2014 | Facebook | (18.5) |
| Banyai, 2017 | Hungary | 5961 | 2015 | Any | 15 -22 (16.6) |
| Barry, 2017 | United States | 113 | 2017 | Any | 14 - 17 (15.3) |
| Blomfield neira, 2014 | Australia | 1819 | NR | Any | 13-17 (14.6) |
| Boer, 2020 | Global | 154,981 | 2017 – 2018 | Any | 11 – 15 (13.5) |
| Bonsaksen, 2023 | Norway | 139,841 | 2021 | Any | 13 – 19 |
| Buda, 2021 | Lithuania | 4191 | 2018 | Any | (13.9) |
| Caner, 2022 | Turkey | 1363 | 2021 | Any | (15.93) |
| Charmaraman, 2022 | United States | 700 | 2017 | Any | 11 - 14 (12.7) |
| Chang, 2019 | Singapore | 303 | 2016 – 2017 | Instagram | 12 – 16 (14.2) |
| Coyne, 2023 | United States | 1231 | 2021 | Any | 10 – 17 (14.5) |
| de Vries, 2019 | Netherlands | 440 | 2016 | Any | 12 – 19 (14.9) |
| Dhir, 2018 | India | 1554 | 2017 | Facebook | 12 – 18 (14.6) |
| Errasti, 2017 | Spain | 503 | NR | Facebook/Twitter | 14 – 17 |
| Fardouly, 2020 | Australia | 528 | 2016 – 2017 | Any | 10 – 12 (11.19) |
| Frison, 2015 | Belgium | 910 | 2013 | Facebook | (15.4) |
| Ghergut, 2021 | Romania | 532 | 2020 | Any | 12 – 15 (13.6) |
| Gurbuz, 2017 | Turkey | 108 | 2014 | Any | 13 – 18 (15.3) |
| Hanprathet, 2015 | Thailand | 972 | NR | Facebook | NR |
| Ho, 2016 | Singapore | 1059 | 2013 | Any | 12 – 19 (14.7) |
| Hosokawa, 2023 | Japan | 161 | 2020 | Any | 12 – 15 |
| Khalil, 2022 | Egypt | 584 | NR | Facebook | 14 – 18 (16.1) |
| Kim, 2020 | South Korea | 209 | 2017 | Any | NR |
| Li, 2017 | China | 1015 | 2013 | Any | NR |
| Lonergan, 2020 | Australia | 4209 | NR | Any | 12 – 18 (15.03) |
| Louragli, 2019 | Morocco | 541 | 2017 | Facebook | 12 – 19 (15.2) |
| Maftei, 2022 | Romania | 488 | NR | Any | 12 – 15 (13.6) |
| Marino, 2020 | Italy | 58976 | 2018 | Any | 11 – 15 (13.5) |
| McLean, 2015 | Australia | 101 | NR | Any | (13.13) |
| Meier, 2014 | United States | 103 | NR | Facebook | 12 – 18 (15.4) |
| Morello, 2023 | Italy | 1195 | 2021 | Any | 14 – 18 (16.2) |
| Morin-Major, 2016 | Canada | 94 | NR | Facebook | 12 – 17 (14.5) |
| Muzi, 2021 | Italy | 62 | 2020 | Any | 12 – 17 (15.43) |
| Naeemi, 2017 | Malaysia | 401 | 2014 | Facebook | 13 – 16 |
| Nesi, 2021 | United States | 243 | 2020 – 2021 | Any | 11 – 18 (15.3) |
| Nesi, 2021 (b) | United States | 639 | 2016 | Any | (17.6) |
| Niu, 2018 | China |  | NR | QZone | 12 – 18 (14.2) |
| Nolan, 2023 | Northern Ireland | 1291 | 2019 – 2020 | Any | 11 – 19 |
| Ophir, 2018 | Israel | 86 | NR | Facebook | 13 – 18 (15.9) |
| Politte-Corn, 2023 | United States | 254 | 2019 – 2021 | Any | 14 – 22 (17.4) |
| Pontes, 2017 | Portugal | 509 | 2015 | Facebook | 10 – 18 (13.0) |
| Skogen, 2021 | Norway | 513 | 2020 | Any | (17.1) |
| Skogen, 2023 | Norway | 3253 | 2020 – 2021 | Any | (17) |
| Swedo, 2020 | United States | 15083 | 2018 | Any | NR |
| Tadena, 2020 | Philippines | 114 | NR | Any | 12 – 17 (14.1) |
| Tao, 2022 | United States | 407 | 2020 – 2021 | Any | 15 – 18 (16.5) |
| Thomas, 2022 | United States | 356 | 2018 | Any | 12 – 18 (16.0) |
| Tian, 2018 | China | 5215 | NR | WeChat/QZone | 10 – 23 (16.2) |
| Tomczyk, 2018 | Bosnia and Herzegovina | 717 | 2017 | Any | (13.0) |
| Valkenburg, 2006 | Netherlands | 881 | NR | CU2 | 10 – 19 (14.8) |
| Walsh, 2020 | Global | 32,884 | 2017 – 2018 | Any | 15 (15.5) |
| Wang, 2018 | China | 365 | 2017 | Any | 14 – 18 (15.9) |
| Watson, 2021 | United States | 441 | NR | Any | 13 – 19 (17.3) |
| Wenninger, 2014 | Germany | 80 | 2013 | Facebook | 13 – 16 (14.9) |
| Wilksch, 2020 | Australia | 996 | 2018 | Facebook, Instagram, Snapchat, Tumblr | (13.1) |
| Woods, 2016 | Scotland | 467 | NR | Any | 11 – 17 |
| Xie, 2018 | China | 1742 | NR | QQ/WeChat | (14.4) |
| Yurdagul, 2021 | Turkey | 493 | NR | Instagram | 14 – 19 (15.9) |
| Yurtdas-Depboylu, 2022 | Turkey | 1232 | 2022 | Any | 13 – 18 (15.7) |
| Ziv, 2016 | Israel | 200 | NR | Facebook | 13 – 26 (17.8) |
| **Cohort** | | | | | |
| Boer, 2020 | Netherlands | 543 | 2015 – 2017 | Any | 12 – 15 (12.9) |
| Boer, 2021 | Netherlands | 2109 | 2016 – 2018 | Any | 10 – 16 (13.1) |
| Chen, 2021 | China | 543 | 2019 – 2020 | Any | (10.32) |
| Cheng, 2023 | Vietnam | 532 | 2021 – 2022 | Any | NR |
| Dumas, 2023 | Canada | 595 | 2020 – 2021 | Any | 14 – 18 (17.8) |
| Dunlop, 2011 | United States | 711 | 2008 – 2009 | Any | 14-22 |
| Fredrick, 2022 | United States | 800 | 2014 – 2016 | Any | 13 – 15 (14.5) |
| Frison, 2016 | Belgium | 1235 | 2013 – 2014 | Facebook | 12 – 19 (14.8) |
| Gingras, 2023 | Canada | 497 | 2017 – 2018 | Any | 13 – 14 (13.7) |
| Li, 2021 | China | 4237 | NR | Any | NR |
| Metzler, 2017 | Germany | 217 | 2013 – 2014 | Facebook | 14 – 18 (16.7) |
| Nesi, 2021 (c) | United States | 687 | 2018 – 2019 | Any | (14.3) |
| Puukko, 2020 | Finland | 2891 | 2014 – 2019 | Any | 13 – 14 |
| Raudsepp, 2019 | Estonia | 397 | 2016 – 2018 | Any | (12.6) |
| Teppers, 2014 | Belgium | 256 | NR | Facebook | 14 – 19 (15.9) |
| Tiggerman, 2017 | Australia | 438 | NR | Facebook | 13 – 15 (13.6) |
| Valkenburg, 2017 | Netherlands | 516 | 2012 – 2014 | Any | 10 – 15 (12.5) |
| Van Den EijnDen, 2018 | Netherlands | 543 | 2015 – 2017 | Any | 12 – 15 (12.9) |
| Vandenbosch, 2016 | Belgium | 1504 | 2010 – 2011 | Any | 12 – 20 (15.4) |
| Vernon, 2017 | Australia | 874 | 2011 – 2014 | Any | (14.4) |
| Wang, 2018 | Belgium | 1188 | 2013 – 2014 | Facebook | (14.3) |
| Winstone, 2022 | England | 2456 | 2019 – 2020 | Any | 13 – 14 (13.2) |

Supplementary Table B: Measures of social media use employed in included studies

| Measurement Tool/Scale | Scale Purpose/Contents | Studies |
| --- | --- | --- |
| **Social Media Use Habits** |  |  |
| Use of Facebook Questionnaire^1^ | 13-item scale with items measuring the frequency of posting, the expression of personal emotions, and the expression of empathy towards other’s emotions | ^1^ |
| Facebook Intensity Scale^2^ | Number of friends, amount of time spent on social media, 6-items to measure the extent to which the participant is emotionally connected to Facebook and the extent to which Facebook is integrated into their daily activities | ^3-6^ |
| Social Networking Activity Intensity Scale (SNAIS)^7^ | 14-item scale assessing overall social networking activity, as well as social use intensity (such as messaging) and entertainment use intensity (such as games) | ^8-10^ |
| Multidimensional Scale of Facebook Use^11^ | A 9-item scale assessing three domains of Facebook use: active private use (such as chatting), active public use (such as posting) and passive use (such as browsing) | ^12,13^ |
| Social Network Use Questionnaire^14^ | Non-validated questionnaire designed to assess various types of SM use including depressive disclosures and type of SM activities | ^14,15^ |
| Facebook Use Questionnaire^16^ | 32-item scale designed to capture various aspects of FB use including frequency, duration, extent, type of content, depth of engagement, and importance | ^16^ |
| SNS Frequency Scale^17^ | 5-item scale designed to assess how often participants use SM for different activities such as making friends, staying in touch, and flirting | ^4^ |
| Attractiveness-Related Use of SNS (MAP-SNS)^18^ | 4-item scale to measure use of social media for attractiveness-related reasons such as “When I think a boy or a girl is good looking after a first meeting, I search for his or her profile on Facebook” | ^18^ |
| Summary of Social Media Use^19^ | 5-item scale measuring social media use in different settings such as at meals, right before bed, or as soon as waking up | ^6^ |
| Passive Social Media Use ^20^ | 6-item scale to measure passive use of social media such as browsing and scrolling | ^21^ |
| Mindfulness Attention Awareness Scale (MAAS)^22^ | 14-item scale designed to assess the degree to which mindful attention is absent in a set of activities, in this case, social media usage | ^23^ |
| Internet Motives Questionnaire for Adolescents (IMQ-A)^24^ | 16-item scale to measure adolescent motives for social media use, including coping, socializing, conformity and enhancement | ^25^ |
| Selfie Practices Scale^26^ | 8-item scale for assessing selfie practices including appearance investment, use of editing and importance of peer feedback | ^26^ |
| Socio-digital Participation Inventory^27^ | 4-item scale measuring frequency of active socially-oriented use of SM such as chatting or sharing content | ^28^ |
| Online Civic Engagement Behavior Construct^29^ | 4-item scale designed to assess engagement in racial justice civic engagement, including posting information and organizing activities | ^30^ |
| General non-validated use measures | Non-validated questions designed to capture aspects of social media use such as posting, liking, sharing photos, active versus passive use, or commenting | ^4,23,26,31-51^ |
| **Addiction-like Measures** | |  |
| Bergen Social Media/Facebook Addiction Scale (BSMAS)^52^ | 6-item self-report questionnaire to measure the extent of problematic/addictive social media usage | ^6,46,53-62^ |
| Social Media Disorder Scale^63^ | A 9-item scale based on the 9 DSM-5 criteria for Internet Gaming Disorder with a clear cut-point for disordered use of social media | ^15,38,39,44,64-70^ |
| Facebook Addiction Scale^71^ | 8-items related to the symptoms of cognitive and behavioral salience, conflict with other activities, euphoria, loss of control, withdrawal, and relapse and reinstatement | ^72^ |
| Social Media Use Urges^73^ | 4-item scale to measure the urge to use social media in variable situations. Originally designed to capture aspects of FOMO | ^6^ |
| Young’s Internet Addiction Scale, Adapted for Social Media^74^ | 20-item scale that measures the presence and severity of Internet dependency, adapted to be suitable for social media specifically. Created by adapting DSM-IV criteria for pathological gambling | ^75^ |
| Facebook Intrusion Questionnaire^76^ | 8-item scale that measures the intrusion of Facebook on daily life, including addiction-like constructs such as withdrawal and cognitive salience | ^77^ |
| Social Media Addiction Scale for Adolescents (SMASA)^78^ | 9-item scale developed to assess social media addiction | ^79^ |
| Problematic Use of Mobile Phones Scale (PUMP), adapted for Social Media^80^ | Participants are first asked which social media site they use the most often, and then asked follow-up questions regarding their use of that site | ^23^ |
| Non-validated scales for SNS addiction | Non-validated questions designed to assess SNS addiction | ^81,82^ |
| **Structural Aspects** |  |  |
| Number of accounts/platforms used | The number of distinct accounts the adolescent has on SM platforms | ^83^ ^64,84^ |
| Number of friends/followers | The number of friends or followers the adolescent has on their SM account | ^1,26,31,43,48,49,85-87^ |
| Number of posts | The number of posts an adolescent has. Includes regular posts, photos, or selfies. | ^26,48,49,85^ |
| Number of likes per post/photo/account | The number of likes the adolescent has received, either per post, per picture, or per account | ^26,31,40,48,49,87,88^ |
| Fake accounts/Alternate accounts | Does the adolescent have any fake SM accounts | ^84^ |
| Following Celebrities/Influencers | Whether or not the adolescent is following celebrities or influencers, and the number/types of influencers followed | ^64,89,90^ |
| **Interactions** |  |  |
| Non-validated measures of SM interactions | Non-validated questions assessing how often SM is used to make friends, start relationships, seek social support, or receive likes/comments from others | ^48,87,88,91^ |
| Use of Facebook/Twitter Questionnaire ^1^ | 12-item questionnaire assessing the amount a user posts on FB, the expression of personal emotions on FB, and the expression of empathy regarding the emotions of others on FB | ^1^ |
| Multidimensional Scale of Perceived Social Support adapted for FB^92^ | 4-item “friends” subscale of the Multidimensional Scale of Perceived Social Support, adapted for FB | ^91^ |
| Online Victimization Scale^93^ | 4-item scale designed to assess past-month experiences with individual and vicarious social media racial discrimination | ^30,34^ |
| Developmental Intergroup Contact Measure^94^ | 8-item scale designed to assess intergroup contact in online settings, adapted to social media specifically | ^30^ |
| Exposure to negative experiences/interactions on SM | Non-validated questionnaire assessing exposure to negative interactions, unwanted contact, and exclusion on SM | ^95^ |
| Tone of interactions | Non-validated questions designed to assess whether interactions on SM are positive or negative | ^48,88^ |
| **Feelings about Social Media** |  |  |
| Frequency of Having a Negative Feeling from Comparison on Facebook Scale | Non-validated questions designed to assess how often a person thinks negatively about themselves from comparisons on SM | ^5,45^ |
| Online Self-Presentation Questionnaire | 4-item scale designed to assess the extent to which participants honestly share their thoughts, feelings, and life events through FB | ^96^ |
| Self-Presentation on Social Media^97^ | 7-item scale designed to assess the participants focus on self-presentation via social media, including retouching photos, curating content, and investment in responses | ^97^ |
| Gratifications derived from social media usage^98^ | 16-item tool designed to measure the extent to which social media information-seeking, socializing, and entertainment on SM provides positive gratification | ^98^ |
| Physical Appearance Comparison Scale, adapted for Social Media^99^ | Four-item scale designed to measure the degree to which an individual compares their physical appearance to others on social media | ^100^ |
| Social Media Comparison | Non-validated measures of social comparison behaviors on social media including social comparison and  appearance comparison | ^15,23,40,100^ |
| Facebook Envy Scale^101^ | 8-item scale to measure how participants compare themselves to others on social media | ^21^ |
| Emotional Responses to Social Media Experiences Scale^102^ | 10-item scale designed to assess the frequency with which positive and negative emotions are experienced during social media use | ^102,103^ |
| Photo Investment Scale^41^ | 8-item scale measuring the effort invested into choosing a selfie for social media, and the response to the selfie | ^36^ |
| Social Media Affinity Scale^104^ |  | ^105^ |
| Social Media Fatigue^106^ | 5-item scale designed to measure social media fatigue defined as Social media fatigue is defined as the tendency to back away from social media usage when they become overwhelmed with too many sites, too many pieces of content, too many friends and contacts and too much time spent keeping up with these connections | ^53^ |
| Non-validated measure of Social Media Fatigue | Non-validated questionnaire designed to assess the degree to which SNS users feel tired or bored from forming, maintaining, and developing online social networking | ^81^ |
| Social media-related body dissatisfaction | Non-validated measure, assessing the degree to which participants feel bad about their body based off social media interactions or use | ^90^ |
| Appearance/photo investment on SM | Non-validated measures of appearance or photo investment, including items like removing pictures if they do not get any/enough comments or likes | ^40,41^ |
| Social Media Use Integration Scale^107^ | 10-item scale of online social media use that measures the integration of the social behavior and daily routines of users, along with the importance of and emotional connection to this use. | ^108^ |
| Non-validated measures of self-presentation on SM | Questions designed to measure the extent to which participants selectively show aspects of themselves on SM | ^87^ |
| Non-validated measures of SM feelings | Pressure to gain likes/followers | ^31^ |

Supplementary Table C: Mental health measures used in included studies

| **Scales Used** | **Number of studies used** | **Study Citation** |
| --- | --- | --- |
| **Depression (n=41)** |  |  |
| Center for Epidemiologic Studies of Depression Scale for Children (CES-DC) | 14 | ^3,5,9,21,30,32,55,62,72,77,85,90,91,96^ |
| Short Mood and Feelings Questionnaire (SMFQ) | 6 | ^26,33,34,40,95,97,102^ |
| Children’s Depression Inventory (CDI) | 3 | ^14,43,46^ |
| Patient Health Questionnaire (PHQ) | 2 | ^23,54^ |
| The Depression Scale (DEPS) | 2 | ^28,53^ |
| Hospital Anxiety and Depression Scale (HADS) | 2 | ^35,108^ |
| Depressive Mood Inventory | 2 | ^15,109^ |
| DSM-5 Checklist | 1 | ^84^ |
| Depression, Anxiety, Stress Scale-21 (DASS-21) | 1 | ^59^ |
| General Health Questionnaire (GHQ-28) | 1 | ^56^ |
| Revised Child Anxiety and Depression Scale (RCADS-25) | 1 | ^8^ |
| Beck Depression Inventory (BDI) | 1 | ^49^ |
| Michigan study of adolescent life transitions | 1 |  |
| Short Depression-Happiness Scale (SDHS) | 1 | ^60^ |
| MINI International Neuropsychiatry Interview for children and adolescents (MINI KID) | 1 | ^57^ |
| ASEBA Youth Self-Report Form questions 11-18 | 1 | ^65^ |
| Depressed mood scale | 1 | ^4^ |
| **Anxiety (n=22)** |  |  |
| Generalized Anxiety Disorder Assessment (GAD-7) | 4 | ^30,58,95,97^ |
| Social Anxiety Scale Short Form (SAS-A) | 3 | ^53,60,90^ |
| DSM-5 Checklist | 1 | ^84^ |
| Depression, Anxiety, Stress Scale-21 (DASS-21) | 1 | ^59^ |
| General Health Questionnaire (GHQ-28) | 1 | ^56^ |
| Revised Children’s Manifest Anxiety Scale (RCMAS-2) | 1 | ^46^ |
| Revised Child Anxiety and Depression Scale (RCADS-25) | 1 | ^8^ |
| State-Trait Anxiety Inventory Short Form (STAI-6) | 1 | ^60^ |
| Hospital Anxiety and Depression Scale (HADS) | 1 | ^108^ |
| Beck Anxiety Inventory Trait (BAIT) | 1 | ^38^ |
| Brief Assessment of Anxiety and PTSD Symptoms | 1 | ^34^ |
| Youth Self Report 11-18 | 1 | ^65^ |
| Social Anxiety Inventory | 1 | ^14^ |
| MINI International Neuropsychiatry Interview for children and adolescents (MINI KID) | 1 | ^57^ |
| Spence Children’s Anxiety Scale | 1 | ^40^ |
| Social Appearance Anxiety Scale | 1 | ^64^ |
| Non-validated measure of anxiety symptoms | 1 | ^81^ |
| **Psychological Complaints/Internalizing Symptoms (n=13)** |  |  |
| HBSC Symptom Checklist | 3 | ^44,68,69^ |
| Strengths and Difficulties Questionnaire (SDQ) | 2 | ^23,25^ |
| General Health Questionnaire (GHQ-28) | 1 | ^56^ |
| Depression, Anxiety, Stress Scale-21 (DASS-21) | 1 | ^61^ |
| Positive and Negative Affect Schedule (PANAS) | 2 | ^21,98^ |
| Youth Pediatric Symptom Checklist (PSC-17) | 1 | ^103^ |
| Brief Symptom Inventory^110^ | 1 | ^31^ |
| ASEBA Youth Self-Report Form 11-18 | 1 | ^65^ |
| Ruminative Response Scale | 1 | ^77^ |
| **ADHD (n=4)** |  |  |
| ADHD-Questionnaire* | 1 | ^39^ |
| DSM-5 Checklist | 1 | ^84^ |
| Youth-Pediatric Symptom Checklist (PSC-17) | 1 | ^103^ |
| ASEBA Youth Self Report Form 11-18 | 1 | ^65^ |
| **Oppositional Defiant Disorder (n=1)** |  |  |
| DSM-5 Checklist (ODD) | 1 | ^84^ |
| **Suicidal Behavior (n=4)** |  |  |
| The Suicidal Ideation Questionnaire-Junior (SIQ-JR) | 1 | ^103^ |
| Suicidal Behaviors Questionnaire | 1 | ^50^ |
| Non-validated measure of suicidal ideation/self-harm | 2 | ^35,51^ |
| **Self-Esteem (n=12)** |  |  |
| Rosenburg Self-Esteem Scale | 7 | ^1,5,43,48,55,77,108^ |
| Self-Perception Profile for Adolescents | 2 | ^47,88^ |
| Inventory of Self-Concept and Self-Confidence | 1 | ^87^ |
| Internet self-efficacy scale | 1 | ^82^ |
| Non-validated measure of self-esteem | 1 | ^4^ |
| **Other Ill-being (n=13)** |  |  |
| Fear of Missing Out Scale (FOMO) | 3 | ^53^ |
| UCLA Loneliness Scale | 3 | ^3,60,84^ |
| Loneliness Scale | 1 | ^13^ |
| Loneliness and Aloneness Scale for Children and Adolescents (LACA) | 1 | ^111^ |
| Short Loneliness Scale | 1 | ^38^ |
| Loneliness and Social Dissatisfaction Questionnaire | 1 | ^102^ |
| Cortisol Levels | 1 | ^43^ |
| Perceived Stress Scale | 1 | ^43^ |
| Depression, Anxiety, Stress Scale-21 (DASS-21) | 1 | ^59^ |
| **Wellbeing/Life Satisfaction (n=16)** |  |  |
| Satisfaction with Life Scale | 4 | ^45,48,70,88^ |
| Cantril's Ladder | 3 | ^44,67,69^ |
| Warwick–Edinburgh Well-being Scale (WEMWBS) | 3 | ^35,95,97^ |
| Student’s Life Satisfaction Scale | 2 | ^3,15^ |
| General Well-being Index | 1 | ^16^ |
| Ryff Psychological Well-being Scale | 1 | ^82^ |
| **Body Image/Body Dissatisfaction/Eating Disorders (n=33)** |  |  |
| Children’s Eating Attitude Test/Eating Attitudes Test (EAT) | 5 | ^8,40,79,83,105^ |
| Body Esteem Scale | 4 | ^23,26,37,40^ |
| Eating Disorders Inventory | 3 | ^41,86,100^ |
| Sociocultural Attitudes Toward Appearance scale | 3 | ^18,41,86^ |
| Objectified Body Consciousness Scale for Adolescents | 2 | ^18,86^ |
| Eating disorder Examination Questionnaire (EDE-Q) | 2 | ^36,83^ |
| Self-Objectification Questionnaire | 2 | ^18,42^ |
| Body Attitude Test (BAT) | 1 | ^12^ |
| Body Image Dissatisfaction Scale (BIDS) | 1 | ^60^ |
| SCOFF Questionnaire | 1 | ^66^ |
| Body Shape Questionnaire | 1 | ^89,105^ |
| Physical Appearance Comparison Scale (PACS) | 1 | ^37^ |
| The Body Image State Scale | 1 |  |
| Physical Appearance Trait Anxiety Scale (PASTAS) | 1 | ^10^ |
| Body Image Scale (BIS) | 1 | ^79^ |
| Male Body Attitudes Scale (MBAS) | 1 | ^100^ |
| Drive for Muscularity Scale | 1 | ^100^ |
| Orthorexia Nervosa Scale (ORTO) | 1 | ^79^ |
| Binge Eating Scale (BES) | 1 | ^65^ |

Supplementary Table D: Assessment of the quality of included studies

| **Study** | **Selection** | **Comparability** | **Outcome** | **Total** |
| --- | --- | --- | --- | --- |
|  | Maximum of 5 stars | Maximum of 2 Stars | Maximum of 3 Stars | 0-10 Stars |
| **Cross Sectional** | | | | |
| Apaolaza, 2014 | ★★ |  | ★★★ | 5 |
| Azhari, 2022 | ★★ |  | ★★★ | 5 |
| Banjanin, 2014 | ★★ |  | ★★★ | 5 |
| Banyai, 2017 | ★★★★ |  | ★★★ | 7 |
| Barry, 2017 | ★ | ★ | ★★★ | 5 |
| Blomfield neira, 2014 | ★★★ | ★ | ★★ | 6 |
| Boer, 2020 | ★★★★ | ★★ | ★★★ | 9 |
| Bonsaksen, 2023 | ★★★★ | ★★ | ★★★ | 9 |
| Buda, 2021 | ★★★★ | ★★ | ★★★ | 9 |
| Caner, 2022 | ★★★ | ★★ | ★★★ | 8 |
| Charmaraman, 2022 | ★★ | ★★ | ★★★ | 7 |
| Chang, 2019 | ★ |  | ★★★ | 4 |
| Coyne, 2023 | ★ | ★★ | ★★ | 5 |
| de Vries, 2019 | ★★ | ★★ | ★★★ | 7 |
| Dhir, 2018 | ★★ |  | ★★★ |  |
| Errasti, 2017 | ★★★ |  | ★★★ | 6 |
| Fardouly, 2020 | ★ | ★★ | ★★★ | 6 |
| Frison, 2015 | ★ | ★★ | ★★★ | 6 |
| Ghergut, 2021 | ★★★ | ★ | ★★★ | 7 |
| Gurbuz, 2017 |  |  |  |  |
| Hanprathet, 2015 | ★★★★★ | ★★ | ★★★ | 10 |
| Ho, 2016 | ★★★ | ★★ | ★★★ | 8 |
| Hosokawa, 2023 | ★★ | ★★ | ★★★ | 7 |
| Khalil, 2022 | ★★★ |  | ★★★ | 6 |
| Kim, 2020 | ★ | ★★ |  | 3 |
| Li, 2017 | ★★★ | ★★ | ★★★ | 8 |
| Lonergan, 2020 | ★★★★★ | ★★ | ★★★ | 10 |
| Louragli, 2019 | ★★★ |  | ★★ | 5 |
| Maftei, 2022 | ★★ | ★★ | ★★★ | 7 |
| Marino, 2020 | ★★★★ | ★★ | ★★★ | 9 |
| McLean, 2015 | ★ |  | ★★★ | 4 |
| Meier, 2014 | ★ | ★ | ★★ | 4 |
| Morello, 2023 | ★★ | ★★ | ★★★ | 7 |
| Morin-Major, 2016 | ★ | ★★ | ★★★ | 6 |
| Muzi, 2021 | ★★★★ | ★ | ★★★ | 8 |
| Naeemi, 2017 | ★★ |  | ★★★ | 5 |
| Nesi, 2021 |  | ★★ | ★★★ | 4 |
| Nesi, 2021 (b) | ★★★★ | ★★ | ★★★ | 9 |
| Niu, 2018 | ★★ | ★★ | ★★ | 6 |
| Nolan, 2023 | ★★★★ | ★★ | ★★★ | 9 |
| Ophir, 2018 | ★★ |  | ★★★ | 5 |
| Pontes, 2017 | ★★★ | ★★ | ★★★ | 8 |
| Skogen, 2021 | ★★ | ★ | ★★★ | 6 |
| Skogen, 2023 | ★★ | ★★ | ★★★ | 7 |
| Swedo, 2021 | ★★★★ | ★★ | ★★★ | 9 |
| Tadena, 2020 | ★★★ |  | ★★★ | 6 |
| Tao, 2022 | ★★★★ | ★★ | ★★★ | 9 |
| Thomas, 2022 | ★★★ | ★★ | ★★★ | 8 |
| Tian, 2018 | ★★★★ | ★★ | ★★★ | 9 |
| Tomczyk, 2018 | ★★ |  | ★★ | 4 |
| Valkenburg, 2006 | ★ | ★★ | ★★★ | 6 |
| Walsh, 2020 | ★★★ | ★★ | ★★★ | 8 |
| Wang, 2018 | ★★ |  | ★★★ | 5 |
| Watson, 2021 | ★★★★ | ★★ | ★★★ | 9 |
| Wenninger, 2014 | ★ | ★★ | ★ | 4 |
| Wilksch, 2020 | ★ | ★ | ★★★ | 5 |
| Woods, 2016 | ★★ |  | ★★ | 4 |
| Xie, 2018 | ★★★ | ★ | ★★★ | 7 |
| Yurdagul, 2021 | ★★★ | ★★ | ★★★ | 8 |
| Yurtdas-Depboylu, 2022 | ★★★ | ★★ | ★★★ | 8 |
| Ziv, 2016 | ★★ |  | ★★ | 4 |
| **Cohort** | | | | |
| Boer, 2020 | ★★★ | ★★ | ★★★ | 8 |
| Boer, 2021 | ★★★ | ★★ | ★★★ | 8 |
| Chen, 2021 | ★★ | ★★ | ★★★ | 7 |
| Cheng, 2023 | ★ |  | ★★ | 3 |
| Dunlop, 2011 | ★★ |  | ★★ | 4 |
| Dumas, 2023 | ★ | ★ | ★★ | 4 |
| Fredrick, 2022 | ★★ | ★★ | ★★ | 6 |
| Frison, 2016 | ★★ | ★★ | ★★★ | 7 |
| Gingras, 2023 | ★★★ | ★ | ★★★ | 7 |
| Li, 2021 | ★★★ | ★★ | ★★★ | 8 |
| Metzler, 2017 | ★★ | ★ | ★★★ | 6 |
| Nesi, 2021 (c) | ★★★ | ★ | ★★★ | 7 |
| Raudsepp, 2019 | ★★★ | ★ | ★★★ | 7 |
| Teppers, 2014 | ★★ | ★★ | ★★★ | 7 |
| Tiggerman, 2017 | ★★ |  | ★★ | 4 |
| Valkenburg, 2017 | ★★ | ★ | ★★★ | 7 |
| Van Den EijnDen, 2018 | ★★★ | ★★ | ★★ | 7 |
| Vandenbosch, 2016 | ★★★ | ★★ | ★★★ | 8 |
| Vernon, 2017 | ★★★ | ★★ | ★★★ | 8 |
| Wang, 2018 | ★★ | ★★ | ★★★ | 7 |
| Winstone, 2022 | ★★ | ★★ | ★★★ | 7 |

**Appendix 1: Preferred Reporting Items for Systematic reviews and Meta-Analyses extension for Scoping Reviews (PRISMA-ScR) Checklist**

| **SECTION** | **ITEM** | **PRISMA-ScR CHECKLIST ITEM** | **REPORTED ON PAGE #** |
| --- | --- | --- | --- |
| **TITLE** | | | |
| Title | 1 | Identify the report as a scoping review. | Title |
| **ABSTRACT** | | | |
| Structured summary | 2 | Provide a structured summary that includes (as applicable): background, objectives, eligibility criteria, sources of evidence, charting methods, results, and conclusions that relate to the review questions and objectives. | Abstract |
| **INTRODUCTION** | | | |
| Rationale | 3 | Describe the rationale for the review in the context of what is already known. Explain why the review questions/objectives lend themselves to a scoping review approach. | Lines 17-35 |
| Objectives | 4 | Provide an explicit statement of the questions and objectives being addressed with reference to their key elements (e.g., population or participants, concepts, and context) or other relevant key elements used to conceptualize the review questions and/or objectives. | Lines 32-35 |
| **METHODS** | | | |
| Protocol and registration | 5 | Indicate whether a review protocol exists; state if and where it can be accessed (e.g., a Web address); and if available, provide registration information, including the registration number. | N/A |
| Eligibility criteria | 6 | Specify characteristics of the sources of evidence used as eligibility criteria (e.g., years considered, language, and publication status), and provide a rationale. | Lines 40-52 |
| Information sources* | 7 | Describe all information sources in the search (e.g., databases with dates of coverage and contact with authors to identify additional sources), as well as the date the most recent search was executed. | Lines 44-46 |
| Search | 8 | Present the full electronic search strategy for at least 1 database, including any limits used, such that it could be repeated. | Appendix 2 |
| Selection of sources of evidence† | 9 | State the process for selecting sources of evidence (i.e., screening and eligibility) included in the scoping review. | Lines 48-73 |
| Data charting process‡ | 10 | Describe the methods of charting data from the included sources of evidence (e.g., calibrated forms or forms that have been tested by the team before their use, and whether data charting was done independently or in duplicate) and any processes for obtaining and confirming data from investigators. | Lines 75-79 |
| Data items | 11 | List and define all variables for which data were sought and any assumptions and simplifications made. | Lines 75-79 |
| Critical appraisal of individual sources of evidence§ | 12 | If done, provide a rationale for conducting a critical appraisal of included sources of evidence; describe the methods used and how this information was used in any data synthesis (if appropriate). | Lines 80-86 |
| Synthesis of results | 13 | Describe the methods of handling and summarizing the data that were charted. | Lines 87-90 |
| **RESULTS** | | | |
| Selection of sources of evidence | 14 | Give numbers of sources of evidence screened, assessed for eligibility, and included in the review, with reasons for exclusions at each stage, ideally using a flow diagram. | Figure 1 |
| Characteristics of sources of evidence | 15 | For each source of evidence, present characteristics for which data were charted and provide the citations. | Table 1, Supplement A |
| Critical appraisal within sources of evidence | 16 | If done, present data on critical appraisal of included sources of evidence (see item 12). | Supplement D |
| Results of individual sources of evidence | 17 | For each included source of evidence, present the relevant data that were charted that relate to the review questions and objectives. | Supplement E |
| Synthesis of results | 18 | Summarize and/or present the charting results as they relate to the review questions and objectives. | Lines 93-302,Table 2 |
| **DISCUSSION** | | | |
| Summary of evidence | 19 | Summarize the main results (including an overview of concepts, themes, and types of evidence available), link to the review questions and objectives, and consider the relevance to key groups. | Lines 305-414 |
| Limitations | 20 | Discuss the limitations of the scoping review process. | Lines 395-404 |
| Conclusions | 21 | Provide a general interpretation of the results with respect to the review questions and objectives, as well as potential implications and/or next steps. | Lines 405-414 |
| **FUNDING** | | | |
| Funding | 22 | Describe sources of funding for the included sources of evidence, as well as sources of funding for the scoping review. Describe the role of the funders of the scoping review. | Click here to enter text. |

JBI = Joanna Briggs Institute; PRISMA-ScR = Preferred Reporting Items for Systematic reviews and Meta-Analyses extension for Scoping Reviews.

* Where *sources of evidence* (see second footnote) are compiled from, such as bibliographic databases, social media platforms, and Web sites.

† A more inclusive/heterogeneous term used to account for the different types of evidence or data sources (e.g., quantitative and/or qualitative research, expert opinion, and policy documents) that may be eligible in a scoping review as opposed to only studies. This is not to be confused with *information sources* (see first footnote).

‡ The frameworks by Arksey and O’Malley (6) and Levac and colleagues (7) and the JBI guidance (4, 5) refer to the process of data extraction in a scoping review as data charting*.*

§ The process of systematically examining research evidence to assess its validity, results, and relevance before using it to inform a decision. This term is used for items 12 and 19 instead of "risk of bias" (which is more applicable to systematic reviews of interventions) to include and acknowledge the various sources of evidence that may be used in a scoping review (e.g., quantitative and/or qualitative research, expert opinion, and policy document).

*From:* Tricco AC, Lillie E, Zarin W, O'Brien KK, Colquhoun H, Levac D, et al. PRISMA Extension for Scoping Reviews (PRISMAScR): Checklist and Explanation. Ann Intern Med. 2018;169:467–473. [doi: 10.7326/M18-0850](http://annals.org/aim/fullarticle/2700389/prisma-extension-scoping-reviews-prisma-scr-checklist-explanation).

**Appendix 2: Search strategy example, optimized for PubMed**

**("Social Media"[Mesh] OR**

“Social media”[tiab] OR

“Media, Social”[tiab] OR

“Social network*”[tiab] OR

“Digital media*”[tiab] OR

“Twitter”[tiab] OR

“Facebook”[tiab] OR

“Instagram”[tiab] OR

“Tumblr”[tiab] OR

“TikTok”[tiab] OR

“Reddit”[tiab]) AND

**(“Adolescent"[Mesh]”** **OR**

“Adolescen*”[tiab] OR

“Youth”[tiab] OR

“Young person*”[tiab] OR

“Young people”[tiab] OR

“Highschooler*”[tiab] OR

**"Child"[Mesh] OR**

“Child*”[tiab] OR

“Teen*”[tiab]) AND

("Systematic Review" [Publication Type] OR

“Systematic Review”[tiab] OR

“Scoping Review”[tiab])

**Appendix 3: Newcastle Ottawa Scale For Cohort Studies**

Note: A study can be awarded a maximum of one star for each numbered item within the Selection and Outcome categories. A maximum of two stars can be given for Comparability

**Selection**

1. Representativeness of the exposed cohort
   1. truly representative of the average adolescent in the target population (random sampling) ★
   2. somewhat representative of the average adolescent in the target population (non-random sampling) ★
   3. Convenience sample or selected group of users eg. nurses, volunteers
   4. no description of the derivation of the cohort
2. Selection of the non-exposed cohort
   1. drawn from the same community as the exposed cohort ★
   2. drawn from a different source
   3. no description of the derivation of the non-exposed cohort
3. Ascertainment of exposure
   1. Objective measure ★
   2. Self-report using a validated measure ★
   3. Self-report using a non-validated measure
   4. no description
4. Demonstration that outcome of interest was not present at start of study
   1. Yes ★
   2. No

**Comparability**

1. Comparability of cohorts on the basis of the design or analysis
   1. study controls for gender ★
   2. study controls for age ★

**Outcome**

1. Assessment of outcome
   1. Self-report using a validated measure ★
   2. Self-report using a non-validated measure
   3. No description
2. Was follow-up long enough for outcomes to occur
   1. Yes ★
   2. No
3. Adequacy of follow up of cohorts
   1. complete follow up - all subjects accounted for ★
   2. subjects lost to follow up unlikely to introduce bias - small number lost >90% follow up, or description provided of those lost ★
   3. follow up rate < 90% and no description of those lost
   4. no statement

**Newcastle Ottawa Scale For Cross-Sectional Studies**

Note: A study can be awarded a maximum of one star for each numbered item within the Selection and Outcome categories. A maximum of two stars can be given for Comparability

**Selection**

1. Representativeness of the cases
   1. Truly representative of the average adolescent in the target population (random sampling) ★
   2. Somewhat representative of the average adolescent in the target population (non-random sampling) ★
   3. Convenience sample or selected group
   4. No description of the sampling strategy
2. Sample size
   1. Justified with sample size calculation ★
   2. Not justified
3. Non-response rate
   1. Response rate is satisfactory (>80%) ★
   2. Differences in respondents and non-respondents characterized/description of non-respondents ★
   3. Sample size is unsatisfactory, and no description of non-respondents ★
4. Ascertainment of exposure
   1. Objective measure ★★
   2. Self-report using a validated measure ★★
   3. Self-report using a non-validated measure, but the measure is available ★
   4. Self-report using a non-validated measure, not available
   5. No description

**Comparability**

1. The potential confounders were investigated by a sub-group analysis or controlled for in multivariate analysis
   1. study controls for gender ★
   2. study controls for age ★

**Outcome**

1. Assessment of outcome
   1. Self-report using a validated measure ★★
   2. Self-report using a non-validated measure, but the measure is available ★
   3. Self-report using a non-validated measure, not available
   4. No description
2. Statistical test
   1. The statisitical test used to analyze the data is clearly described and appropriate ★
   2. The statistical test is not appropriate, not described, or incomplete

1. Errasti J, Amigo I, Villadangos M. Emotional Uses of Facebook and Twitter: Its Relation With Empathy, Narcissism, and Self-Esteem in Adolescence. *Psychological Reports*. 2017/12/01 2017;120(6):997-1018. doi:10.1177/0033294117713496

2. Ellison NB, Steinfield C, Lampe C. The benefits of Facebook 'friends:' Social capital and college students' use of online social network sites. *Journal of Computer-Mediated Communication*. 2007;12(4):1143-1168. doi:10.1111/j.1083-6101.2007.00367.x

3. Tian Y, Zhang S, Wu R, Wang P, Gao F, Chen Y. Association Between Specific Internet Activities and Life Satisfaction: The Mediating Effects of Loneliness and Depression. *Front Psychol*. 2018;9:1181. doi:10.3389/fpsyg.2018.01181

4. Blomfield neira CJ, Barber BL. Social networking site use: Linked to adolescents' social self‐concept, self‐esteem, and depressed mood. *Australian Journal of Psychology*. 2014/03/01 2014;66(1):56-64. doi:10.1111/ajpy.12034

5. Niu GF, Luo YJ, Sun XJ, et al. Qzone use and depression among Chinese adolescents: A moderated mediation model. *J Affect Disord*. Apr 15 2018;231:58-62. doi:10.1016/j.jad.2018.01.013

6. Tomczyk Ł, Selmanagic-Lizde E. Fear of Missing Out (FOMO) among youth in Bosnia and Herzegovina — Scale and selected mechanisms. *Children and Youth Services Review*. 2018/05/01/ 2018;88:541-549. doi:<https://doi.org/10.1016/j.childyouth.2018.03.048>

7. Li J, Lau JT, Mo PK, et al. Validation of the Social Networking Activity Intensity Scale among Junior Middle School Students in China. *PLoS One*. 2016;11(10):e0165695. doi:10.1371/journal.pone.0165695

8. Ghergut A, Maftei A, Ana G. The Light and the Dark Side of Social Media Use: Depression, Anxiety, and Eating Attitudes among Adolescents. *Psihologija*. 07/19 2021;55doi:10.2298/PSI210516001G

9. Li JB, Feng LF, Wu AMS, et al. Roles of Psychosocial Factors on the Association Between Online Social Networking Use Intensity and Depressive Symptoms Among Adolescents: Prospective Cohort Study. *J Med Internet Res*. Sep 21 2021;23(9):e21316. doi:10.2196/21316

10. Maftei A. How do social networks, controlling parenting, and interpersonal sensitivity contribute to adolescents' appearance anxiety? *Current Psychology*. 10/07 2022;42doi:10.1007/s12144-022-03839-9

11. Frison E, Eggermont S. Toward an Integrated and Differential Approach to the Relationships Between Loneliness, Different Types of Facebook Use, and Adolescents’ Depressed Mood. *Communication Research*. 2020;47(5):701-728. doi:10.1177/0093650215617506

12. de Vries DA, Vossen HGM, van der Kolk-van der Boom P. Social Media and Body Dissatisfaction: Investigating the Attenuating Role of Positive Parent-Adolescent Relationships. *J Youth Adolesc*. Mar 2019;48(3):527-536. doi:10.1007/s10964-018-0956-9

13. Wang K, Frison E, Eggermont S, Vandenbosch L. Active public Facebook use and adolescents' feelings of loneliness: Evidence for a curvilinear relationship. *J Adolesc*. Aug 2018;67:35-44. doi:10.1016/j.adolescence.2018.05.008

14. Akkın Gürbüz HG, Demir T, Gökalp Özcan B, Kadak MT, Poyraz BÇ. Use of social network sites among depressed adolescents. *Behaviour & Information Technology*. 2017;36(5):517-523. doi:10.1080/0144929X.2016.1262898

15. Boer M, Stevens GWJM, Finkenauer C, de Looze ME, van den Eijnden RJJM. Social media use intensity, social media use problems, and mental health among adolescents: Investigating directionality and mediating processes. *Computers in Human Behavior*. 2021;116doi:10.1016/j.chb.2020.106645

16. Ziv I, Kiasi M. Facebook's Contribution to Well-being among Adolescent and Young Adults as a Function of Mental Resilience. *J Psychol*. 2016;150(4):527-41. doi:10.1080/00223980.2015.1110556

17. Lenhart A, Madden M. Teens, privacy and online social networks. 2007;

18. Vandenbosch L, Eggermont S. The Interrelated Roles of Mass Media and Social Media in Adolescents’ Development of an Objectified Self-Concept:A Longitudinal Study. *Communication Research*. 2016;43(8):1116-1140. doi:10.1177/0093650215600488

19. Hetz PR, Dawson CL, Cullen TA. Social media use and the fear of missing out (FoMO) while studying abroad. *Journal of research on technology in education*. 2015;47(4):259-272.

20. Wang J-L, Gaskin J, Rost DH, Gentile DA. The reciprocal relationship between passive social networking site (SNS) usage and users’ subjective well-being. *Social Science Computer Review*. 2018;36(5):511-522.

21. Cheng W, Nguyen DN, Nguyen PNT. The association between passive social network usage and depression/negative emotions with envy as a mediator. *Scientific Reports*. 2023/06/21 2023;13(1):10097. doi:10.1038/s41598-023-37185-y

22. Brown KW, West AM, Loverich TM, Biegel GM. Assessing adolescent mindfulness: validation of an adapted Mindful Attention Awareness Scale in adolescent normative and psychiatric populations. *Psychol Assess*. Dec 2011;23(4):1023-33. doi:10.1037/a0021338

23. Coyne SM, Weinstein E, Sheppard JA, et al. Analysis of Social Media Use, Mental Health, and Gender Identity Among US Youths. *JAMA Netw Open*. Jul 3 2023;6(7):e2324389. doi:10.1001/jamanetworkopen.2023.24389

24. Bischof-Kastner C, Kuntsche E, Wolstein J. Identifying problematic Internet users: development and validation of the Internet Motive Questionnaire for Adolescents (IMQ-A). *J Med Internet Res*. Oct 9 2014;16(10):e230. doi:10.2196/jmir.3398

25. Morello L, Scaini S, Caputi M, Frisiello A, Forresi B. Motivations for using social media, adverse experiences and adolescent emotional difficulties in the pandemic context. *Current Psychology: A Journal for Diverse Perspectives on Diverse Psychological Issues*. 2024;43(20):18851-18862. doi:10.1007/s12144-023-05008-y

26. Nesi J, Choukas-Bradley S, Maheux AJ, et al. Selfie Appearance Investment and Peer Feedback Concern: Multi-Method Investigation of Adolescent Selfie Practices and Adjustment. *Psychol Pop Media Cult*. Oct 2021;10(4):488-499. doi:10.1037/ppm0000342

27. Hietajärvi L, Seppa J, Hakkarainen K. Dimensions of adolescents' socio-digital participation. *QWERTY-Interdisciplinary Journal of Technology, Culture and Education*. 2016;11(2):79-98.

28. Puukko K, Hietajärvi L, Maksniemi E, Alho K, Salmela-Aro K. Social Media Use and Depressive Symptoms-A Longitudinal Study from Early to Late Adolescence. *Int J Environ Res Public Health*. Aug 14 2020;17(16)doi:10.3390/ijerph17165921

29. Warren AM, Sulaiman A, Jaafar NI. Social media effects on fostering online civic engagement and building citizen trust and trust in institutions. *Government information quarterly*. 2014;31(2):291-301.

30. Tao X, Fisher CB. Exposure to Social Media Racial Discrimination and Mental Health among Adolescents of Color. *J Youth Adolesc*. Jan 2022;51(1):30-44. doi:10.1007/s10964-021-01514-z

31. Dumas TM, Tremblay PF, Ellis W, Millett G, Maxwell-Smith MA. Does pressure to gain social media attention have consequences for adolescents’ friendship closeness and mental health? A longitudinal examination of within-person cross-lagged relations. *Computers in Human Behavior*. 2023/03/01/ 2023;140:107591. doi:<https://doi.org/10.1016/j.chb.2022.107591>

32. Fredrick SS, Nickerson AB, Livingston JA. Adolescent social media use: Pitfalls and promises in relation to cybervictimization, friend support, and depressive symptoms. *Journal of Youth and Adolescence*. 2022;51(2):361-376. doi:10.1007/s10964-021-01561-6

33. Politte-Corn M, Nick EA, Kujawa A. Age-related differences in social media use, online social support, and depressive symptoms in adolescents and emerging adults. *Child Adolesc Ment Health*. Nov 2023;28(4):497-503. doi:10.1111/camh.12640

34. Thomas A, Jing M, Chen H-Y, Crawford EL. Taking the good with the bad?: Social Media and Online Racial Discrimination Influences on Psychological and Academic Functioning in Black and Hispanic Youth. *Journal of Youth and Adolescence*. 2023/02/01 2023;52(2):245-257. doi:10.1007/s10964-022-01689-z

35. Winstone L, Mars B, Haworth CMA, Heron J, Kidger J. Adolescent social media user types and their mental health and well-being: Results from a longitudinal survey of 13-14-year-olds in the United Kingdom. *JCPP Adv*. Jun 2022;2(2):e12071. doi:10.1002/jcv2.12071

36. Lonergan AR, Bussey K, Fardouly J, et al. Protect me from my selfie: Examining the association between photo-based social media behaviors and self-reported eating disorders in adolescence. *Int J Eat Disord*. May 2020;53(5):485-496. doi:10.1002/eat.23256

37. Chang L, Li P, Loh RSM, Chua THH. A study of Singapore adolescent girls' selfie practices, peer appearance comparisons, and body esteem on Instagram. *Body Image*. Jun 2019;29:90-99. doi:10.1016/j.bodyim.2019.03.005

38. Azhari A, Toms Z, Pavlopoulou G, Esposito G, Dimitriou D. Social media use in female adolescents: Associations with anxiety, loneliness, and sleep disturbances. *Acta Psychol (Amst)*. Sep 2022;229:103706. doi:10.1016/j.actpsy.2022.103706

39. Boer M, van den Eijnden R, Boniel-Nissim M, et al. Adolescents' Intense and Problematic Social Media Use and Their Well-Being in 29 Countries. *J Adolesc Health*. Jun 2020;66(6s):S89-s99. doi:10.1016/j.jadohealth.2020.02.014

40. Fardouly J, Magson NR, Rapee RM, Johnco CJ, Oar EL. The use of social media by Australian preadolescents and its links with mental health. *J Clin Psychol*. Jul 2020;76(7):1304-1326. doi:10.1002/jclp.22936

41. McLean SA, Paxton SJ, Wertheim EH, Masters J. Photoshopping the selfie: Self photo editing and photo investment are associated with body dissatisfaction in adolescent girls. *Int J Eat Disord*. Dec 2015;48(8):1132-40. doi:10.1002/eat.22449

42. Meier EP, Gray J. Facebook photo activity associated with body image disturbance in adolescent girls. *Cyberpsychol Behav Soc Netw*. Apr 2014;17(4):199-206. doi:10.1089/cyber.2013.0305

43. Morin-Major JK, Marin MF, Durand N, Wan N, Juster RP, Lupien SJ. Facebook behaviors associated with diurnal cortisol in adolescents: Is befriending stressful? *Psychoneuroendocrinology*. Jan 2016;63:238-46. doi:10.1016/j.psyneuen.2015.10.005

44. Boer M, Stevens G, Finkenauer C, van den Eijnden R. Attention Deficit Hyperactivity Disorder-Symptoms, Social Media Use Intensity, and Social Media Use Problems in Adolescents: Investigating Directionality. *Child Dev*. Jul 2020;91(4):e853-e865. doi:10.1111/cdev.13334

45. Frison E, Eggermont S. "Harder, Better, Faster, Stronger": Negative Comparison on Facebook and Adolescents' Life Satisfaction Are Reciprocally Related. *Cyberpsychol Behav Soc Netw*. Mar 2016;19(3):158-64. doi:10.1089/cyber.2015.0296

46. Gingras MP, Brendgen M, Beauchamp MH, et al. Adolescents and Social Media: Longitudinal Links Between Types of Use, Problematic Use and Internalizing Symptoms. *Res Child Adolesc Psychopathol*. Nov 2023;51(11):1641-1655. doi:10.1007/s10802-023-01084-7

47. Valkenburg PM, Koutamanis M, Vossen HGM. The concurrent and longitudinal relationships between adolescents' use of social network sites and their social self-esteem. *Comput Human Behav*. Nov 2017;76:35-41. doi:10.1016/j.chb.2017.07.008

48. Wenninger H, Krasnova H, Buxmann P. *Activity Matters: Investigating the Influence of Facebook on Life Satisfaction of Teenage Users*. 2014.

49. The digital footprints of adolescent depression, social rejection and victimization of bullying on Facebook. doi:10.1016/j.chb.2018.09.025. Elsevier Science; 2019.

50. Swedo EA, Beauregard JL, de Fijter S, et al. Associations Between Social Media and Suicidal Behaviors During a Youth Suicide Cluster in Ohio. *J Adolesc Health*. Feb 2021;68(2):308-316. doi:10.1016/j.jadohealth.2020.05.049

51. Dunlop SM, More E, Romer D. Where do youth learn about suicides on the Internet, and what influence does this have on suicidal ideation? *J Child Psychol Psychiatry*. Oct 2011;52(10):1073-80. doi:10.1111/j.1469-7610.2011.02416.x

52. Andreassen CS, Torsheim T, Brunborg GS, Pallesen S. Development of a Facebook Addiction Scale. *Psychol Rep*. Apr 2012;110(2):501-17. doi:10.2466/02.09.18.Pr0.110.2.501-517

53. Dhir A, Yossatorn Y, Kaur P, Chen S. Online social media fatigue and psychological wellbeing—A study of compulsive use, fear of missing out, fatigue, anxiety and depression. *International Journal of Information Management*. 2018/06/01/ 2018;40:141-152. doi:<https://doi.org/10.1016/j.ijinfomgt.2018.01.012>

54. Watson JC, Prosek EA, Giordano AL. Distress Among Adolescents: An Exploration of Mattering, Social Media Addiction, and School Connectedness. *Journal of Psychoeducational Assessment*. 2022/02/01 2021;40(1):95-107. doi:10.1177/07342829211050536

55. Bányai F, Zsila Á, Király O, et al. Problematic Social Media Use: Results from a Large-Scale Nationally Representative Adolescent Sample. *PLoS One*. 2017;12(1):e0169839. doi:10.1371/journal.pone.0169839

56. Hanprathet N, Manwong M, Khumsri J, Yingyeun R, Phanasathit M. Facebook Addiction and Its Relationship with Mental Health among Thai High School Students. *J Med Assoc Thai*. Apr 2015;98 Suppl 3:S81-90.

57. Khalil SA, Kamal H, Elkholy H. The prevalence of problematic internet use among a sample of Egyptian adolescents and its psychiatric comorbidities. *Int J Soc Psychiatry*. Mar 2022;68(2):294-300. doi:10.1177/0020764020983841

58. Louragli I, Ahami AOT, Khadmaoui A, Aboussaleh Y, Lamrani AC. BEHAVIORAL ANALYSIS OF ADOLESCENT’S STUDENTS ADDICTED TO FACEBOOK AND ITS IMPACT ON PERFORMANCE AND MENTAL HEALTH. 2019:

59. Pontes HM. Investigating the differential effects of social networking site addiction and Internet gaming disorder on psychological health. *J Behav Addict*. Dec 1 2017;6(4):601-610. doi:10.1556/2006.6.2017.075

60. Yurdagül C, Kircaburun K, Emirtekin E, Wang P, Griffiths MD. Psychopathological Consequences Related to Problematic Instagram Use Among Adolescents: The Mediating Role of Body Image Dissatisfaction and Moderating Role of Gender. *International Journal of Mental Health and Addiction*. 2021/10/01 2021;19(5):1385-1397. doi:10.1007/s11469-019-00071-8

61. Chen IH, Chen CY, Pakpour AH, et al. Problematic internet-related behaviors mediate the associations between levels of internet engagement and distress among schoolchildren during COVID-19 lockdown: A longitudinal structural equation modeling study. *J Behav Addict*. Feb 10 2021;10(1):135-148. doi:10.1556/2006.2021.00006

62. Raudsepp L, Kais K. Longitudinal associations between problematic social media use and depressive symptoms in adolescent girls. *Prev Med Rep*. Sep 2019;15:100925. doi:10.1016/j.pmedr.2019.100925

63. van den Eijnden RJJM, Lemmens JS, Valkenburg PM. The Social Media Disorder Scale. *Computers in Human Behavior*. 2016/08/01/ 2016;61:478-487. doi:<https://doi.org/10.1016/j.chb.2016.03.038>

64. Caner N, Efe YS, Başdaş Ö. The contribution of social media addiction to adolescent LIFE: Social appearance anxiety. *Curr Psychol*. 2022;41(12):8424-8433. doi:10.1007/s12144-022-03280-y

65. Muzi S, Sansò A, Pace CS. What's Happened to Italian Adolescents During the COVID-19 Pandemic? A Preliminary Study on Symptoms, Problematic Social Media Usage, and Attachment: Relationships and Differences With Pre-pandemic Peers. *Front Psychiatry*. 2021;12:590543. doi:10.3389/fpsyt.2021.590543

66. Nolan E, Bunting L, McCartan C, et al. Prevalence of probable eating disorders and associated risk factors: An analysis of the Northern Ireland Youth Wellbeing Survey using the SCOFF. *Br J Clin Psychol*. Mar 2023;62(1):180-195. doi:10.1111/bjc.12401

67. Buda G, Lukoševičiūtė J, Šalčiūnaitė L, Šmigelskas K. Possible Effects of Social Media Use on Adolescent Health Behaviors and Perceptions. *Psychol Rep*. Jun 2021;124(3):1031-1048. doi:10.1177/0033294120922481

68. Marino C, Lenzi M, Canale N, et al. Problematic social media use: associations with health complaints among adolescents. *Ann Ist Super Sanita*. Oct-Dec 2020;56(4):514-521. doi:10.4415/ann_20_04_16

69. Walsh SD, Sela T, De Looze M, et al. Clusters of Contemporary Risk and Their Relationship to Mental Well-Being Among 15-Year-Old Adolescents Across 37 Countries. *J Adolesc Health*. Jun 2020;66(6s):S40-s49. doi:10.1016/j.jadohealth.2020.02.012

70. van den Eijnden R, Koning I, Doornwaard S, van Gurp F, Ter Bogt T. The impact of heavy and disordered use of games and social media on adolescents' psychological, social, and school functioning. *J Behav Addict*. Sep 1 2018;7(3):697-706. doi:10.1556/2006.7.2018.65

71. Koc M, Gulyagci S. Facebook addiction among Turkish college students: the role of psychological health, demographic, and usage characteristics. *Cyberpsychol Behav Soc Netw*. Apr 2013;16(4):279-84. doi:10.1089/cyber.2012.0249

72. Li JB, Lau JTF, Mo PKH, et al. Insomnia partially mediated the association between problematic Internet use and depression among secondary school students in China. *J Behav Addict*. Dec 1 2017;6(4):554-563. doi:10.1556/2006.6.2017.085

73. Abel JP, Buff CL, Burr SA. Social media and the fear of missing out: Scale development and assessment. *Journal of Business & Economics Research*. 2016;14(1)

74. Young KS. Internet addiction: The emergence of a new clinical disorder. *CyberPsychology & Behavior*. 1998;1(3):237-244. doi:10.1089/cpb.1998.1.237

75. Vernon L, Modecki KL, Barber BL. Tracking Effects of Problematic Social Networking on Adolescent Psychopathology: The Mediating Role of Sleep Disruptions. *J Clin Child Adolesc Psychol*. Mar-Apr 2017;46(2):269-283. doi:10.1080/15374416.2016.1188702

76. Elphinston RA, Noller P. Time to face it! Facebook intrusion and the implications for romantic jealousy and relationship satisfaction. *Cyberpsychol Behav Soc Netw*. Nov 2011;14(11):631-5. doi:10.1089/cyber.2010.0318

77. Wang P, Wang X, Wu Y, et al. Social networking sites addiction and adolescent depression: A moderated mediation model of rumination and self-esteem. *Personality and Individual Differences*. 2018/06/01/ 2018;127:162-167. doi:<https://doi.org/10.1016/j.paid.2018.02.008>

78. Eksi H, Özgenel M, Canpolat Ö. Ergenler için Sosyal Medya Bağımlılığı Ölçeği (ESMBÖ): Geçerlik ve Güvenirlik Çalışması -- Social Media Addiction Scale for Adolescents: Validity and Reliability Study. 07/25 2019;6:629-662. doi:10.15805/addicta.2019.6.3.0086

79. Yurtdaş Depboylu G, Kaner G, Özçakal S. The association between social media addiction and orthorexia nervosa, eating attitudes, and body image among adolescents. *Eating and Weight Disorders - Studies on Anorexia, Bulimia and Obesity*. 12/23 2022;27doi:10.1007/s40519-022-01521-4

80. Stockdale LA, Coyne SM. Bored and online: Reasons for using social media, problematic social networking site use, and behavioral outcomes across the transition from adolescence to emerging adulthood. *J Adolesc*. Feb 2020;79:173-183. doi:10.1016/j.adolescence.2020.01.010

81. Kim MR, Oh JW, Huh BY. Analysis of Factors Related to Social Network Service Addiction Among Korean High School Students. *J Addict Nurs*. Jul/Sep 2020;31(3):203-212. doi:10.1097/jan.0000000000000350

82. Naeemi S, Tamam E. The Relationship Between Emotional Dependence on Facebook and Psychological Well-Being in Adolescents Aged 13–16. *Child Indicators Research*. 2017/12/01 2017;10(4):1095-1106. doi:10.1007/s12187-016-9438-3

83. Wilksch SM, O'Shea A, Ho P, Byrne S, Wade TD. The relationship between social media use and disordered eating in young adolescents. *Int J Eat Disord*. Jan 2020;53(1):96-106. doi:10.1002/eat.23198

84. Barry CT, Sidoti CL, Briggs SM, Reiter SR, Lindsey RA. Adolescent social media use and mental health from adolescent and parent perspectives. *J Adolesc*. Dec 2017;61:1-11. doi:10.1016/j.adolescence.2017.08.005

85. Banjanin N, Banjanin N, Dimitrijevic I, Pantic I. Relationship between internet use and depression: Focus on physiological mood oscillations, social networking and online addictive behavior. *Computers in Human Behavior*. 2015/02/01/ 2015;43:308-312. doi:<https://doi.org/10.1016/j.chb.2014.11.013>

86. Tiggemann M, Slater A. Facebook and body image concern in adolescent girls: A prospective study. *International Journal of Eating Disorders*. 2017/01/01 2017;50(1):80-83. doi:<https://doi.org/10.1002/eat.22640>

87. Metzler A, Scheithauer H. The Long-Term Benefits of Positive Self-Presentation via Profile Pictures, Number of Friends and the Initiation of Relationships on Facebook for Adolescents' Self-Esteem and the Initiation of Offline Relationships. *Front Psychol*. 2017;8:1981. doi:10.3389/fpsyg.2017.01981

88. Valkenburg PM, Peter J, Schouten AP. Friend networking sites and their relationship to adolescents' well-being and social self-esteem. *Cyberpsychol Behav*. Oct 2006;9(5):584-90. doi:10.1089/cpb.2006.9.584

89. Hosokawa R, Kawabe K, Nakachi K, Soga J, Horiuchi F, Ueno S-i. Effects of social media on body dissatisfaction in junior high school girls in Japan. *Eating Behaviors*. 2023/01/01/ 2023;48:101685. doi:<https://doi.org/10.1016/j.eatbeh.2022.101685>

90. Charmaraman L, Richer AM, Liu C, Lynch AD, Moreno MA. Early Adolescent Social Media-Related Body Dissatisfaction: Associations with Depressive Symptoms, Social Anxiety, Peers, and Celebrities. *J Dev Behav Pediatr*. Jun-Jul 01 2021;42(5):401-407. doi:10.1097/dbp.0000000000000911

91. Frison E, Eggermont S. The impact of daily stress on adolescents’ depressed mood: The role of social support seeking through Facebook. *Computers in Human Behavior*. 2015/03/01/ 2015;44:315-325. doi:<https://doi.org/10.1016/j.chb.2014.11.070>

92. Dahlem NW, Zimet GD, Walker RR. The Multidimensional Scale of Perceived Social Support: a confirmation study. *J Clin Psychol*. Nov 1991;47(6):756-61. doi:10.1002/1097-4679(199111)47:6<756::aid-jclp2270470605>3.0.co;2-l

93. Tynes B, Rose C, Williams D. The development and validation of the online victimization scale for adolescents. *Cyberpsychology*. 01/01 2010;4

94. Park H, Gönültaş S, Mulvey KL, Killen M, Ruck MD. Male Adolescents' and Young Adults' Evaluations of Interracial Exclusion in Offline and Online Settings. *Cyberpsychol Behav Soc Netw*. Oct 2019;22(10):641-647. doi:10.1089/cyber.2019.0102

95. Skogen JC, Andersen AIO, Finserås TR, Ranganath P, Brunborg GS, Hjetland GJ. Commonly reported negative experiences on social media are associated with poor mental health and well-being among adolescents: results from the "LifeOnSoMe"-study. *Front Public Health*. 2023;11:1192788. doi:10.3389/fpubh.2023.1192788

96. Xie X, Wang X, Zhao F, Lei L, Niu G, Wang P. Online Real-Self Presentation and Depression among Chinese Teens: Mediating Role of Social Support and Moderating Role of Dispositional Optimism. *Child Indicators Research*. 2018/10/01 2018;11(5):1531-1544. doi:10.1007/s12187-017-9484-5

97. Skogen JC, Hjetland GJ, Bøe T, Hella RT, Knudsen AK. Through the Looking Glass of Social Media. Focus on Self-Presentation and Association with Mental Health and Quality of Life. A Cross-Sectional Survey-Based Study. *Int J Environ Res Public Health*. Mar 23 2021;18(6)doi:10.3390/ijerph18063319

98. Apaolaza V, He J, Hartmann P. The effect of gratifications derived from use of the social networking site Qzone on Chinese adolescents’ positive mood. *Computers in Human Behavior*. 2014/12/01/ 2014;41:203-211. doi:<https://doi.org/10.1016/j.chb.2014.09.029>

99. Thompson JK, Heinberg L, Tantleff-Dunn S. The physical appearance comparison scale. *The Behavior Therapist*. 1991;14:174.

100. Ho SS, Lee EWJ, Liao Y. Social Network Sites, Friends, and Celebrities: The Roles of Social Comparison and Celebrity Involvement in Adolescents’ Body Image Dissatisfaction. *Social Media + Society*. 2016/07/01 2016;2(3):2056305116664216. doi:10.1177/2056305116664216

101. Tandoc Jr EC, Ferrucci P, Duffy M. Facebook use, envy, and depression among college students: Is facebooking depressing? *Computers in human behavior*. 2015;43:139-146.

102. Nesi J, Rothenberg WA, Bettis AH, et al. Emotional Responses to Social Media Experiences Among Adolescents: Longitudinal Associations with Depressive Symptoms. *J Clin Child Adolesc Psychol*. Nov-Dec 2022;51(6):907-922. doi:10.1080/15374416.2021.1955370

103. Nesi J, Burke TA, Extein J, et al. Social media use, sleep, and psychopathology in psychiatrically hospitalized adolescents. *J Psychiatr Res*. Dec 2021;144:296-303. doi:10.1016/j.jpsychires.2021.10.014

104. Gerlich R, Browning L, Westermann L. The Social Media Affinity Scale: Implications For Education. *Contemporary Issues in Education Research (CIER)*. 11/15 2010;3:35. doi:10.19030/cier.v3i11.245

105. Tadena S, Kang SR, Kim SJ. The Influence of Social Media Affinity on Eating Attitudes and Body Dissatisfaction in Philippine Adolescents. *Child Health Nurs Res*. Jan 2020;26(1):121-129. doi:10.4094/chnr.2020.26.1.121

106. Bright LF, Kleiser SB, Grau SL. Too much Facebook? An exploratory examination of social media fatigue. *Computers in Human Behavior*. 2015/03/01/ 2015;44:148-155. doi:<https://doi.org/10.1016/j.chb.2014.11.048>

107. Jenkins-Guarnieri MA, Wright SL, Johnson B. Development and validation of a social media use integration scale. *Psychology of Popular Media Culture*. 2013;2(1):38-50. doi:10.1037/a0030277

108. Woods HC, Scott H. #Sleepyteens: Social media use in adolescence is associated with poor sleep quality, anxiety, depression and low self-esteem. *J Adolesc*. Aug 2016;51:41-9. doi:10.1016/j.adolescence.2016.05.008

109. Bonsaksen T, Steigen AM, Stea TH, Kleppang AL, Lien L, Leonhardt M. Negative social media-related experiences and lower general self-efficacy are associated with depressive symptoms in adolescents. *Front Public Health*. 2022;10:1037375. doi:10.3389/fpubh.2022.1037375

110. Derogatis LR, Melisaratos N. The brief symptom inventory: an introductory report. *Psychological medicine*. 1983;13(3):595-605.

111. Teppers E, Luyckx K, Klimstra TA, Goossens L. Loneliness and Facebook motives in adolescence: a longitudinal inquiry into directionality of effect. *J Adolesc*. Jul 2014;37(5):691-9. doi:10.1016/j.adolescence.2013.11.003
